# Supplementary material for: Syncytial death mediated by oncolytic rVSV-NDV dynamically activates immunogenic apoptosis and necroptosis in human lung cancer cells
Source: Mol Ther Oncol. 2025 Aug 5;33(3):201027. doi: 10.1016/j.omton.2025.201027 (PMC12390950; doi:10.1016/j.omton.2025.201027)
Supplement: Document S1. Figures S1–S8 and Tables S1 and S2 [file mmc1.pdf]

**OMTON, Volume *33***

# Supplemental information

# Syncytial death mediated by oncolytic rVSV-NDV dynamically activates immunogenic apoptosis and necroptosis in human lung cancer cells

**Fabian Kortum, Nina Hartmann, Alexander Bryan, Sonja Glauß, and Jennifer Altomonte**

## Supplemental Tables

**Table S1.** Single guide RNA sequences and targeted exons for CRISPR/Cas9-mediated gene knockout

| Gene target (human) | Ensembl # | sgRNA sequences 5' – 3' | target |
|---|---|---|---|
| Caspase 3 | ENST00000308394 | AGGAATGACATCTCGGTCTG | Exon 5 |
| | | ATGTCGATGCAGCAAACCTC | Exon 5 |
| | | GTCGCTTTGTGCCATGCTGA | Exon 8 |
| Caspase 8 | ENST00000673742 | CCCTCAAGTTCCTGAGCCTG | Exon 2 |
| | | CGGGGATACTGTCTGATCAT | Exon 7 |
| | | CAAACGAGATATATCCCGGA | Exon 8 |
| MLKL | ENST00000308807 | TTGAAGCATATTATCACCCT | Exon 2 |
| | | GCATATTATCACCCTTGGCC | Exon 2 |
| | | TTAGCTTTGGAATCGTCCTC | Exon 9 |
| RIPK1 | ENST00000259808 | GTGTTCCAGGATCGACCTTC | Exon 11 |
| | | TCTACTACATGGCGCCCGAG | Exon 5 |
| | | AGCACCTGAATGACGTCAAC | Exon 5 |
| RIPK3 | ENST00000216274 | CCGCCCCCTTGGTGTCCATC | Exon 2 |
| | | CCTTGGTGTCCATCGAGGAA | Exon 2 |
| | | TCACTCGTGTACGAAGCAGT | Exon 6 |
| NTC1 (from [44]) | / | ACGGAGGCTAAGCGTCGCAA | / |
| NTC2 (from [44]) | / | CGCTTCCGCGGCCCGTTCAA | / |

**Table S2.** Forward and reverse primer sequences

| Primer name | Application | Sequence 5' – 3' | Expected fragment [bp] |
|---|---|---|---|
| RIPK3_exon2_fwd | Primer for CRISPR/Cas9 knockout validation (flanking exon 2 of RIPK3) | CCAGCCTGATGTCGTGCGTCAA | 450 |
| RIPK3_exon2_rev | | ATTCAGGCCCCAGAGCACAGTG | |
| RIPK3_exon6_fwd | Primer for CRISPR/Cas9 knockout validation (flanking exon 6 of RIPK3) | GGATCCTTAACCCCAGGTGCCCTT | 460 |
| RIPK3_exon6_rev | | AGGGACAGCAGAGTGGAGTGCA | |
| MLKL_exon9_fwd | Primer for CRISPR/Cas9 knockout validation (flanking exon 9 of MLKL) | TCAAACTCCTCCAAGGACCACC | 488 |
| MLKL_exon9_rev | | GCCACCAGCTTGCGGATCTTCT | |
| MLKL_exon2_fwd | Primer for CRISPR/Cas9 knockout validation (flanking exon 2 of MLKL) | TGGACCAGCTCTTCCCAGCTACA | 436 |
| MLKL_exon2_rev | | ATCTCCCCATTAGCCTCCTCCAGG | |

Abbreviations: fwd (forward); rev (reverse); bp (base pairs)

## Supplemental Figures

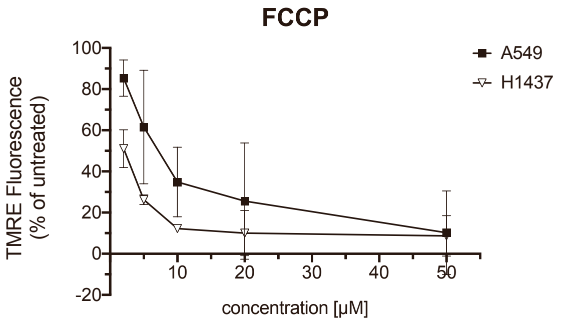


**Figure S1: MOMP induced by the uncoupling agent FCCP in A549 and H1437 cells**

Permeabilization of the outer mitochondrial membrane (MOMP) was mimicked by the uncoupling agent, FCCP at 2, 5, 10, 20, 50 µM. FCCP was added for 10 min, and mitochondria were then stained with TMRE fluorescent dye. Data are shown as % fluorescence reduction relative to untreated cells.

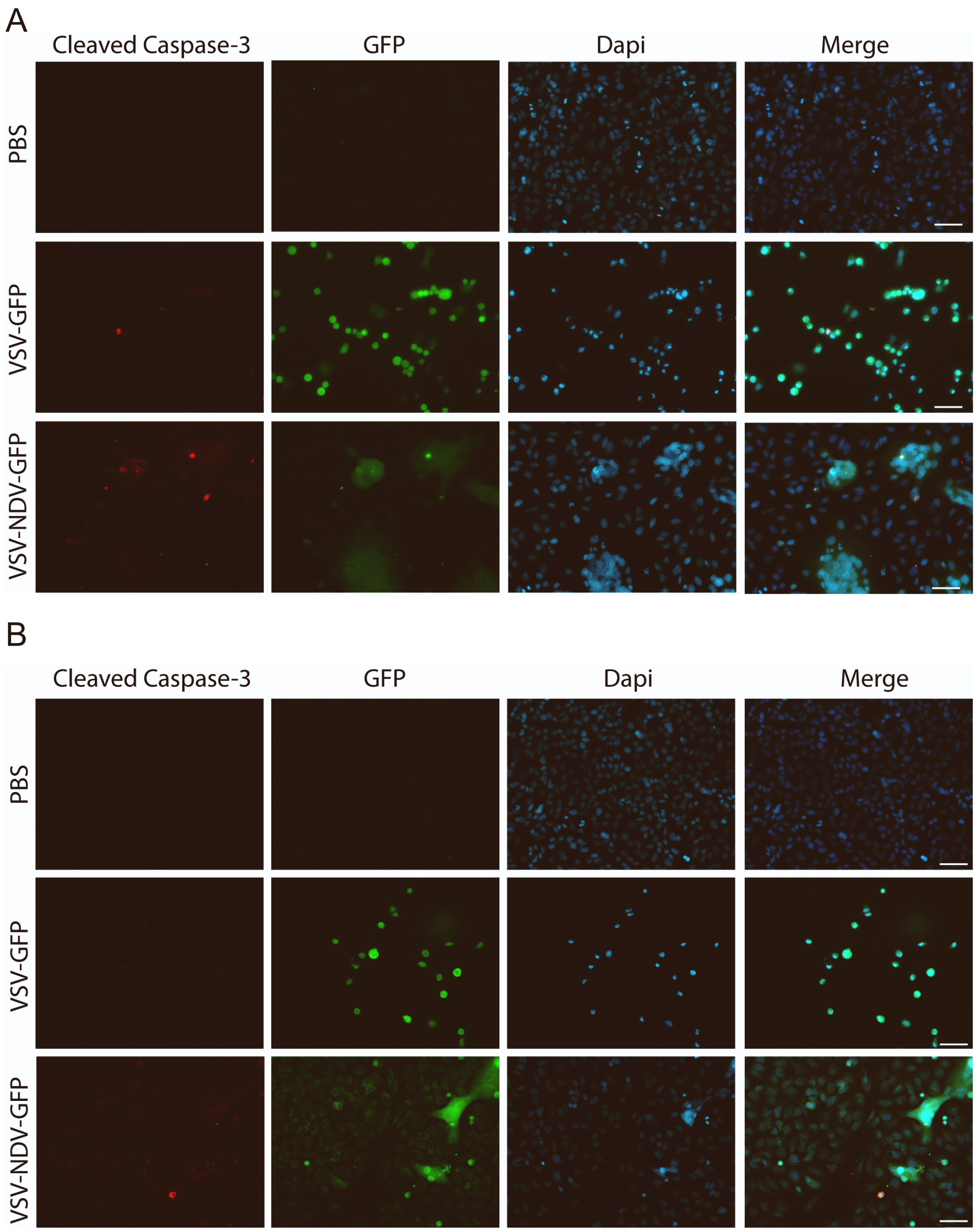


**Figure S2: Cleaved Caspase-3 in VSV- or VSV-NDV-infected A549 cells.** A549 cells were infected in chamber slides at an MOI of 0.01 with rVSV-GFP or rVSV-NDV-GFP or left uninfected. (A) After 24 or (B) 48 hours, cells were fixed and subjected to immunofluorescent staining for cleaved caspase-3. Nuclei were visualized by DAPI staining. Cells were analyzed by fluorescence microscopy, and representative images were captured at 200x magnification. Scale bars represent 50µm.

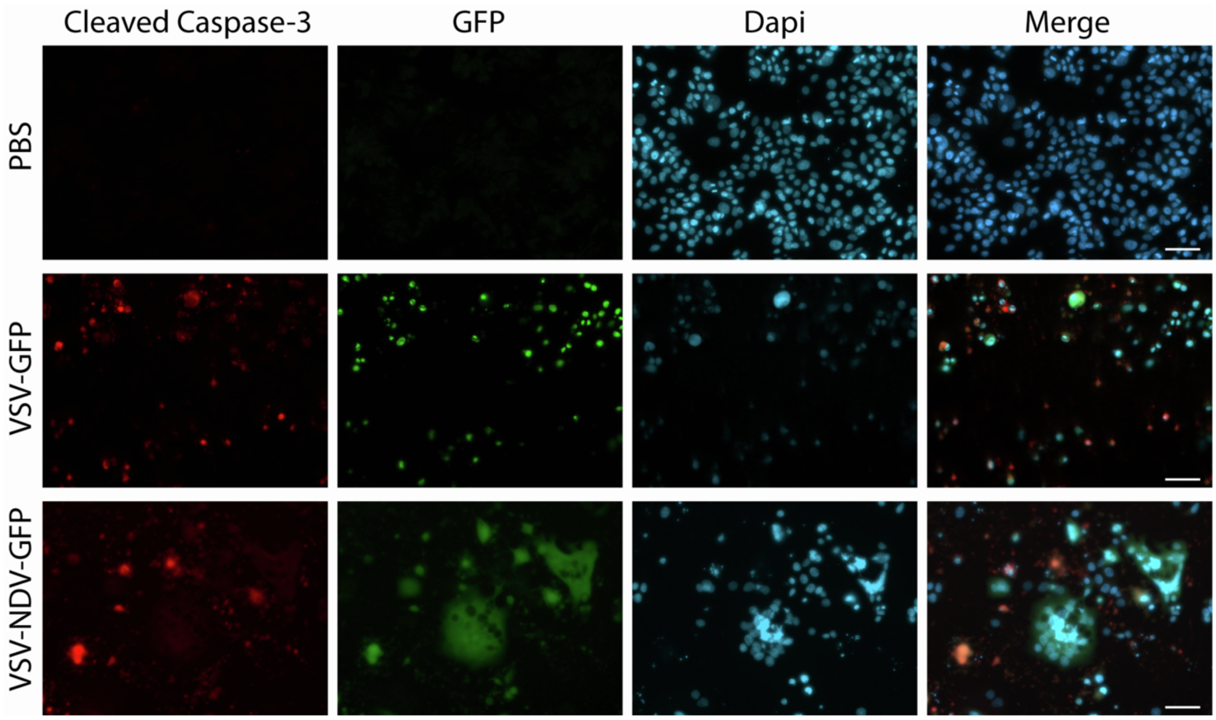


**Figure S3: Cleaved Caspase-3 in VSV- or VSV-NDV-infected H1437 cells.** H1437 cells were infected in chamber slides at an MOI of 0.01 with rVSV-GFP or rVSV-NDV-GFP or left uninfected. After 24 hours, cells were fixed and subjected to immunofluorescent staining for cleaved caspase-3. Nuclei were visualized by DAPI staining. Cells were analyzed by fluorescence microscopy, and representative images were captured at 200x magnification. Scale bars represent 50µm.

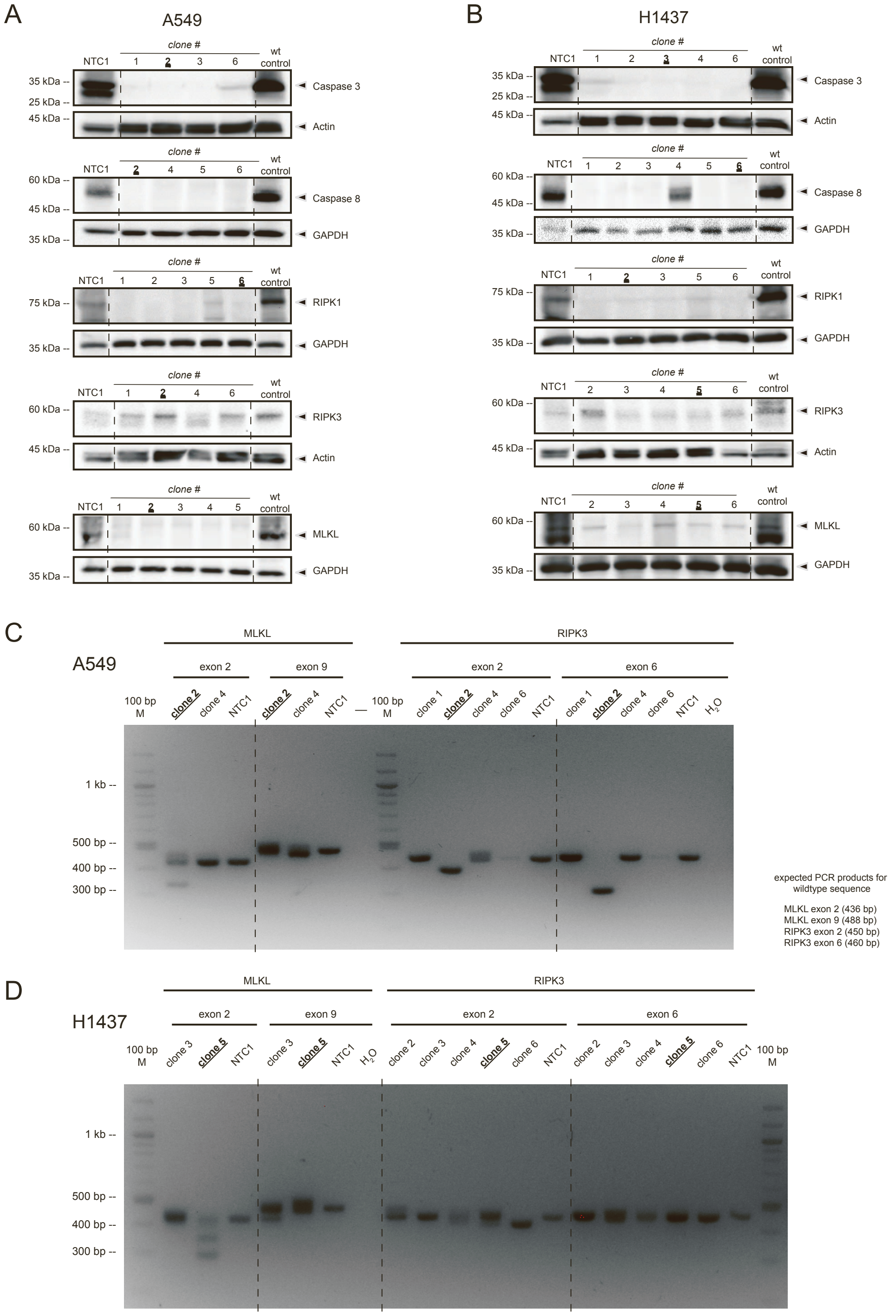


**Figure S4: Western blot and exon PCR validation of CRISPR/Cas9 gene knockouts in A549 and H1437 single cell clones**

Puromycin-resistant single-cell clones for each target gene were isolated by limiting dilution, and successful gene knockout in (**A**) A549 and (**B**) H1437 cells was validated by Western blot analysis and compared to wildtype cells (wt control) or NTC1 controls. Representative blots of n = 1 biological replicate are shown. Arrow-heads on the left indicate the expected protein

bands. RIPK3$^{-/-}$ and MLKL$^{-/-}$ CRISPR/Cas9 gene knockouts in (C) A549 and (D) H1437 single-cell clones were analyzed by PCR over the sgRNA-targeted exons and compared to wildtype or NTC1 cells. Expected PCR product sizes for the non-mutated exons of wildtype MLKL, or wildtype RIPK3 sequence are indicated in (C, right). Knockout clones selected for subsequent experiments are highlighted in red. M indicates the DNA size marker: 100 bp ladder (NEB); kb = kilobases, bp = base pairs.

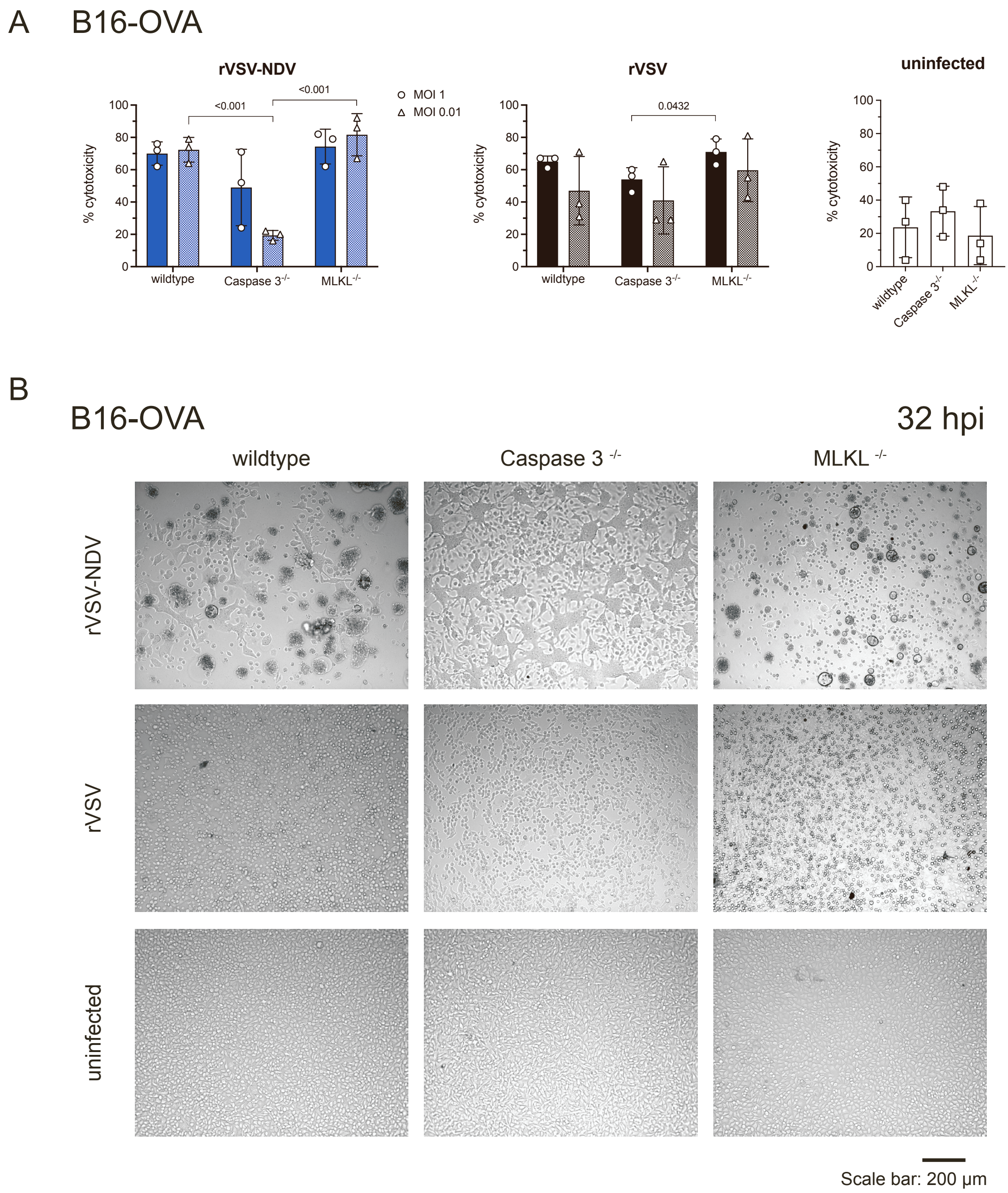


**Figure S5: Impact of genetic deletion of downstream apoptosis or necroptosis effectors on rVSV- or rVSV-NDV-mediated oncolysis in B16-OVA cells**

B16-OVA wildtype, caspase $3^{-/-}$, or $MLKL^{-/-}$ cells were infected at an MOI of 0.01 with either rVSV-NDV-GFP or rVSV-GFP, or left uninfected. (A) Cell death was measured using the LDH assay at 32 hpi, and data are expressed as the mean % cytotoxicity relative to maximum release control (100 % cytotoxicity) +/- SD values from n = 3 biological replicates. Statistical significance was calculated using one-way ANOVA with Tukey's multiple comparisons test and p values are indicated. (B) Oncolysis progression was recorded at 32 hpi by brightfield

microscopy. Representative images of n = 3 independent experiments and images adjusted to similar contrast and brightness are shown.

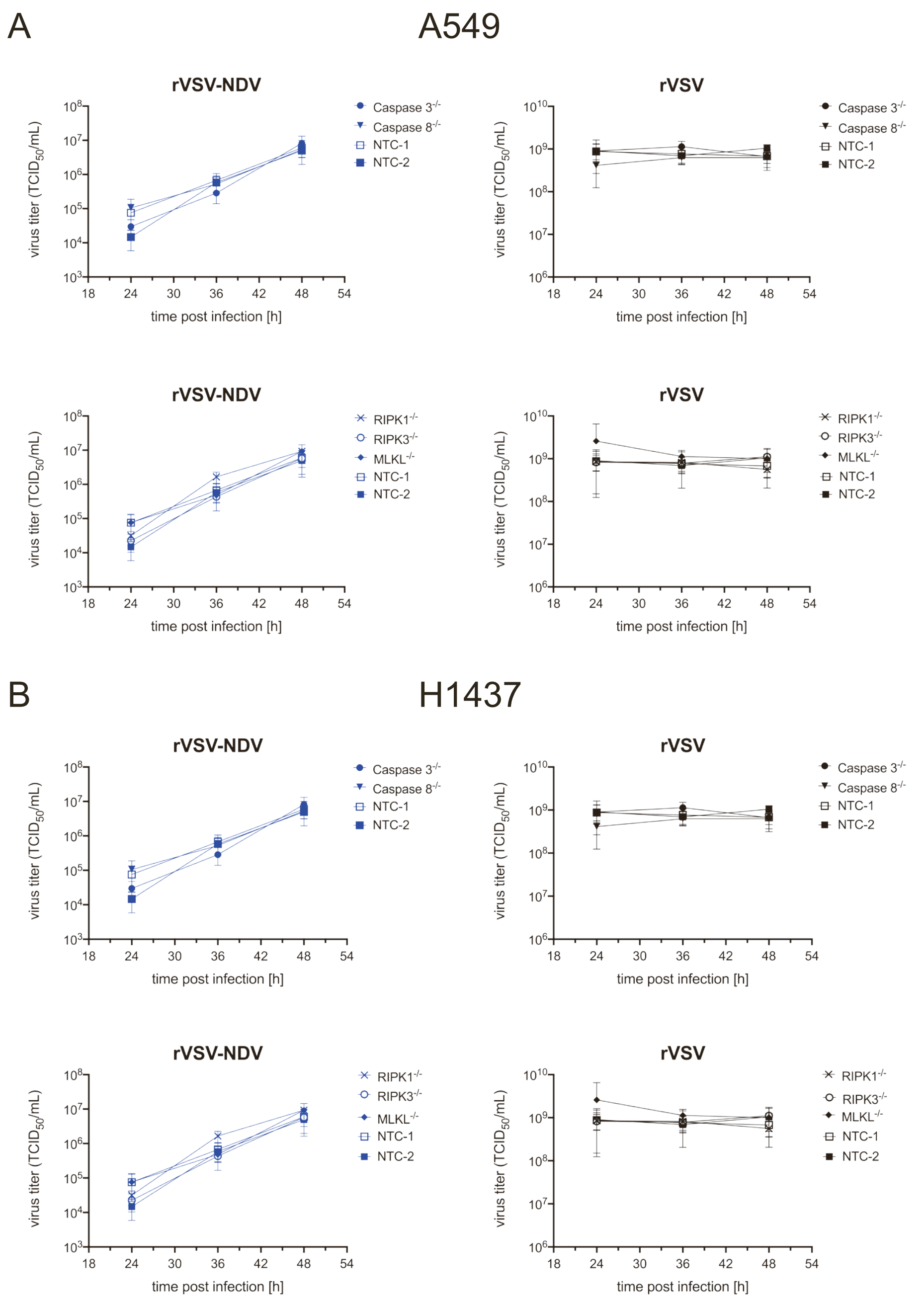


**Figure S6: Time course of oncolytic virus replication in A549 and H1437 knockout cell lines**

(**A**) A549 and (**B**) H1437 knockout clones were infected at an MOI of 0.01, and changes in viral replication were determined by measuring the extracellular virus concentration at 24, 36, and 48 hpi by $TCID_{50}$ assay. For simplicity, graphs are split into a top panel (caspase $3^{-/-}$ and caspase $8^{-/-}$) and a bottom panel (RIPK$1^{-/-}$, RIPK$^{-/-}$, MLKL$^{-/-}$). Virus concentrations of NTC1 and NTC2 are shown in both panels for better side-by-side comparison. Mean +/- SD values of n = 3 independent biological replicates are shown.

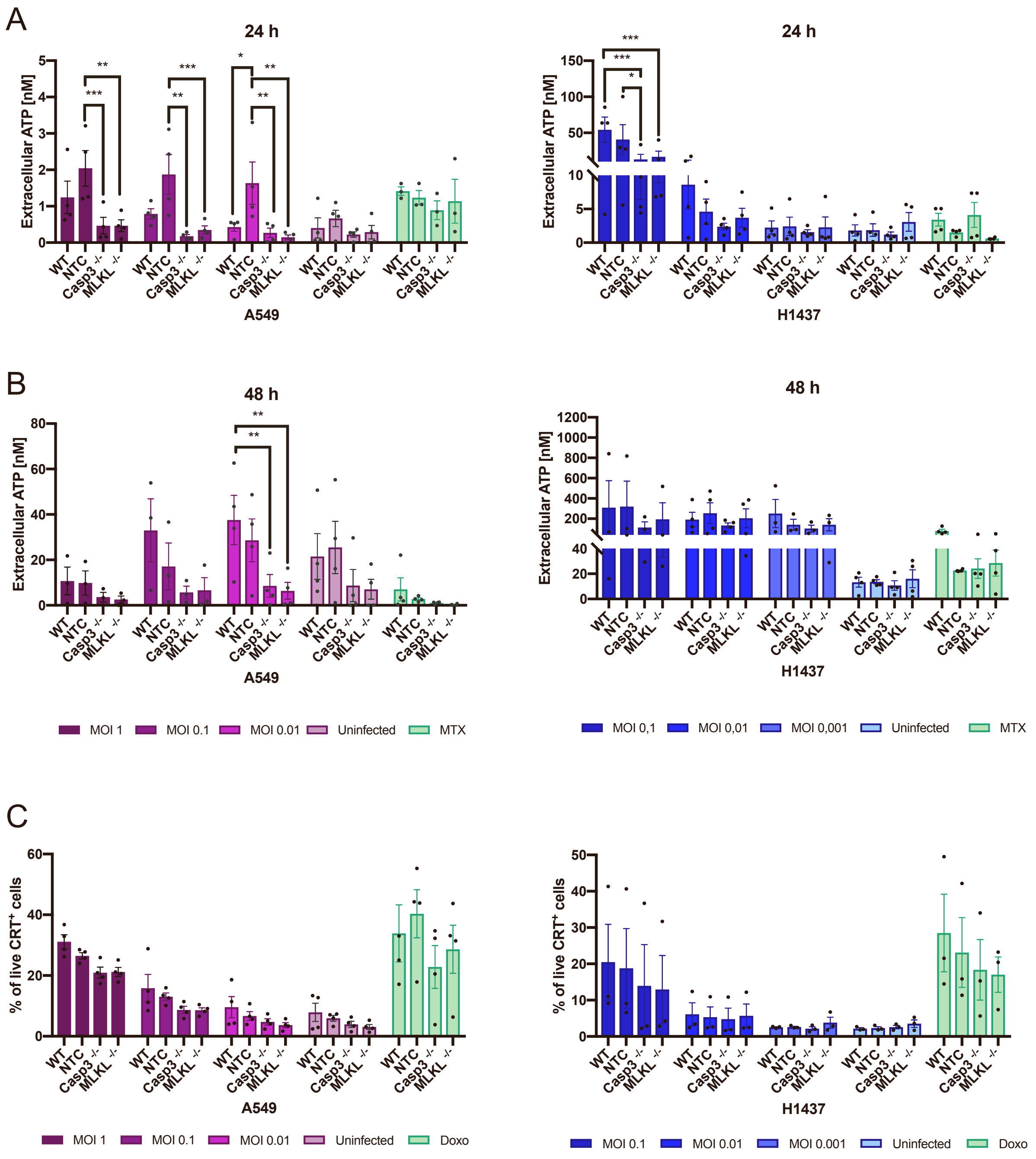


**Figure S7. Immunogenic cell death markers in wildtype versus Caspase-3 and MLKL knockout cells.** A549 and H1437 cells were infected at a range of MOIs with rVSV-NDV, or treated with MTX or doxorubicin as ICD inducer control. (A) Extracellular ATP quantified from supernatants of OV-infected A549 and H1437 cells at 24 and (B) 48 hpi using a commercial ATP Bioluminescent Assay kit. (C) Flow cytometry-based quantification of calreticulin cell surface exposure in OV-infected A549 (A) and H1437 (D) cells at 24 hpi was calculated as % CRT positive cells among live cells and represented as mean (+/- SD) values of n = 3

independent biological replicates. Statistical significance was tested using one-way ANOVA with Tukey's multiple comparisons test. Plotted are mean (SD) values of n = 4 independent biological replicates.

A

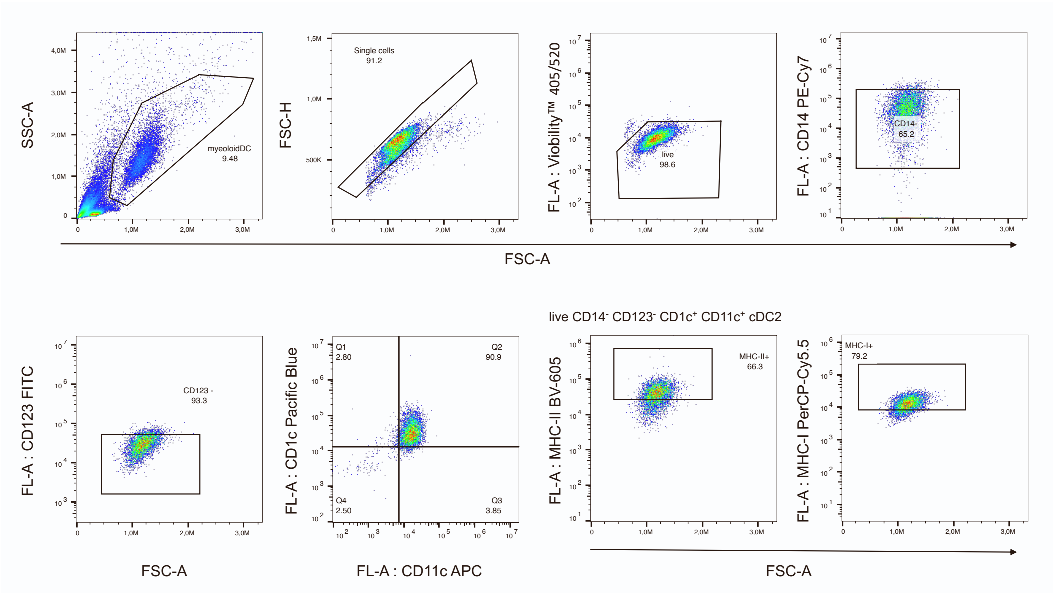


B

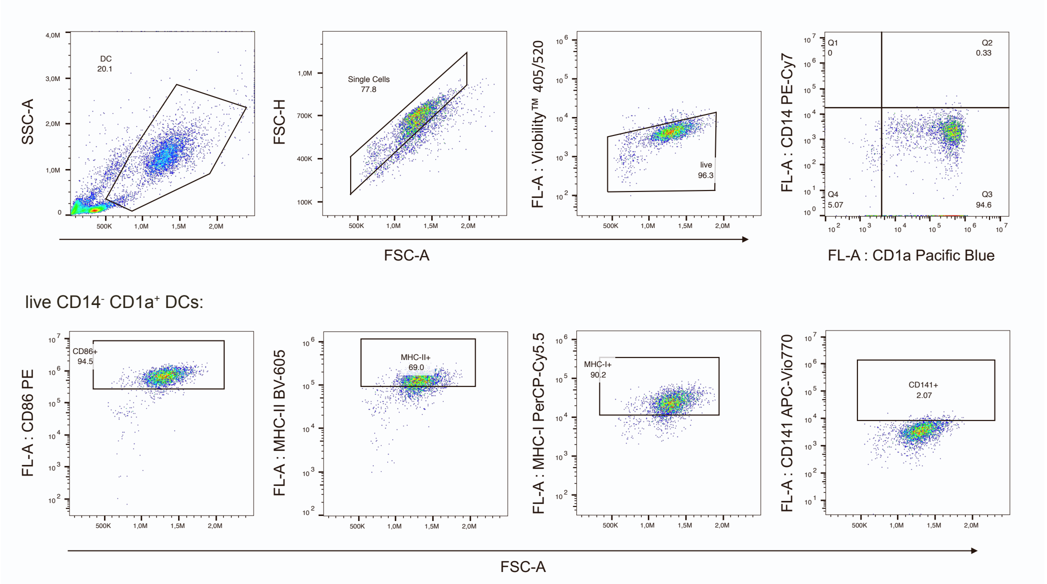


**Figure S8: Exemplified gating strategy for *in vitro*-differentiated human cDC2 subtype DCs, and cDC2 activation after cocktail stimulation**

(A) Human PBMCs from a healthy donor were analyzed 6-days post GM-CSF + IL-4 stimulation by flow cytometry and characterized as cDC2 subtype cells using antibodies against CD1c, CD11c, CD14, CD123, MHC-I and MHC-II. (B) *In vitro*-differentiated cDC2 cells

(live, CD14$^-$, CD1a$^+$) were cocktail-stimulated, and DC activation (MHC-I, MHC-II, CD86) was measured by flow cytometry. CD141 was used to discriminate cDC2 (CD141$^-$) from cDC1 (CD141$^+$) subtypes. (A and B) Representative scatter plots from FlowJo exemplifying the gating strategy for one human donor each are shown. FSC-A = forward scatter area, SSC-A = sideward scatter area, FSC-H = forward scatter height, FL-A = fluorescence channel area.
