# Supplementary material for: Syncytial death mediated by oncolytic rVSV-NDV dynamically activates immunogenic apoptosis and necroptosis in human lung cancer cells
Source: Mol Ther Oncol. 2025 Aug 5;33(3):201027. doi: 10.1016/j.omton.2025.201027 (PMC12390950; doi:10.1016/j.omton.2025.201027)
Supplement: Document S2. Article plus supplemental information [file mmc2.pdf]

Molecular Therapy
Oncology
Original Article

Syncytial death mediated by oncolytic rVSV-NDV dynamically activates immunogenic apoptosis and necroptosis in human lung cancer cells

Fabian Kortum,[1] Nina Hartmann,[1] Alexander Bryan,[1] Sonja Glauß,[1] and Jennifer Altomonte[1]

[1]TUM School of Medicine - Clinical Department of Internal Medicine II, TUM University Hospital, 81675 Munich, Germany

Recent work has indicated that oncolytic virotherapy leads to immunogenic cell death (ICD) as an important mechanism of action; however, the underlying cell death pathways leading to ICD have been less explored. Our previous data demonstrated that chimeric oncolytic recombinant vesicular stomatitis virus-Newcastle disease virus (rVSV-NDV) has a strong immune-stimulating potential that seems to be mediated by immunogenic syncytial oncolysis. In this work, we aimed to investigate the role of apoptosis and necroptosis in mediating syncytial cell death. In human lung cancer cell lines (A549 and H1437), we demonstrate that fusogenic rVSV-NDV and the parental virus, rVSV, both dynamically engage apoptosis and necroptosis signaling to mediate oncolysis. Genetic deletion of key death regulators (caspase-3, caspase-8, RIPK1, RIPK3, and MLKL) by CRISPR-Cas9 further illustrated a cell line-dependent flexibility to switch to alternative, non-apoptotic or non-necroptotic pathways, while maintaining viral replication and net oncolysis. Interestingly, genetic deletion of caspase-3 prolonged the syncytial phenotype and delayed their collapse, suggesting a crucial role of caspases during syncytial death. *In vitro* coculture experiments further revealed that rVSV-NDV-derived oncolysates induced significantly higher levels of differentiation and activation of human monocyte-derived dendritic cells than non-fusogenic rVSV-infected oncolysates. This illustrates the superior immunogenic potential inherent in fusogenic oncolytic viruses and warrants their further preclinical evaluation as next-generation, immune-modulating oncolytic virotherapies.

INTRODUCTION

As a novel class of cancer immunotherapy, replication-competent oncolytic viruses (OVs) provoke a unique, dual attack on tumor cells. OVs directly infect, replicate in, and destroy malignant cells due to their intrinsic or engineered tumor-specificity. Secondary to the oncolytic effect, OVs elicit an immunogenic cell death (ICD) that releases a multitude of pro-inflammatory factors to empower host anti-tumor immunity, which has the potential to trigger a durable line of defense against cancer.[1] Our group has recently introduced an engineered hybrid OV, called rVSV-NDV, consisting of the recombinant vesicular stomatitis virus (rVSV) backbone, in which the glycoprotein (VSV-G) has been replaced by the hemagglutinin-neuraminidase and a modified fusion protein of oncolytic Newcastle disease virus (NDV/F_{3aa}(L289A)).[2] Compared with its parental viruses, rVSV-NDV demonstrated superior safety, oncolytic efficacy, and improved immunogenicity in murine melanoma and hepatocellular carcinoma (HCC) models.[2–4] Owing to the exposure of the pseudotyped NDV envelope proteins on the surface of infected cells, rVSV-NDV exhibits the unique feature of spreading via cell-to-cell fusion (syncytia formation).

It has previously been shown that syncytial death is a potent mode of oncolysis, primarily due to a more efficient dissemination to neighboring uninfected tumor cells, facilitated debulking of tissue-dense tumors (bystander killing),[5] and the potent release of immune-stimulatory factors that serve to augment a systemic and cellular anti-tumor immune response, which is often more prevalent compared with that achieved by nonfusogenic viruses.[6–9] We and others have established that, by modifying rVSV strains to express fusogenic membrane proteins, vectors not only attain the ability to spread via syncytium formation but also substantially enhance the immunogenicity of the tumor.[2,10,11] The hybrid vector, rVSV-NDV, demonstrated enhanced release of canonical ICD markers from HCC cells, achieved promising synergism with adoptive cell therapy, and sensitized tumor cells to immune checkpoint inhibitor therapy.[2–4] However, syncytial cell death is considered an atypical cellular phenotype,[6] and a mechanistic link between fusogenicity and immunogenicity, as well as the underlying cell death mechanism, are still poorly defined.

rVSV has been well characterized to activate and manipulate caspase-dependent, intrinsic and extrinsic apoptosis, at different stages, to sustain viral replication and oncolysis via cell rounding and shrinkage.[12–14] Data obtained from various NDV-infected tumor cell lines indicate that, beside the engagement of apoptosis, fusogenic oncolysis also involves additional death pathways, like autophagy,

Received 11 March 2025; accepted 30 July 2025;
https://doi.org/10.1016/j.omton.2025.201027.

Correspondence: Jennifer Altomonte, TUM School of Medicine and Health - Clinical Department of Internal Medicine II, TUM University Hospital, 81675 Munich, Germany.
E-mail: jennifer.altomonte@tum.de

Published by Elsevier Inc. on behalf of The American Society of Gene and Cell Therapy.
This is an open access article under the CC BY license (http://creativecommons.org/licenses/by/4.0/).

pyroptosis, and necroptosis.[15–19] Necroptosis is a caspase-independent, regulated form of necrosis that involves necrosome complexes (consisting of receptor interacting serine/threonine protein kinase [RIPK] 1, RIPK3, and mixed-lineage kinase domain-like [MLKL]) and phospho-MLKL-mediated membrane pores to induce typical cell swelling until rupture.[20] As a novel oncolytic hybrid vector, the cell death pathway(s) underlying rVSV-NDV-mediated fusogenic oncolysis are yet unknown. While numerous cell death pathways are likely at play, we aimed to focus on the contribution of the two most common cell death pathways, apoptosis and necroptosis, to the fusogenic cytopathic effects (CPEs) of rVSV-NDV in comparison with that of its parental non-fusogenic rVSV. Necroptosis has especially gained attention as a relevant pathway in viral oncolysis and for mediating direct stimulation of an ICD. Oncolytic adenovirus had recently been characterized in myeloma models to induce a caspase-independent, likely necroptotic, type of cell death associated with high ICD marker expression and elevated antigen presentation via major histocompatibility complex (MHC) class I and MHC class II.[21] Moreover, a current strategy followed in the field is the direct targeting and stimulation of RIP3-mediated necroptosis. Kim and colleagues[22] have demonstrated that a polymer-based RIPK3 gene delivery led to RIPK3/MLKL-mediated necroptosis stimulation, increased cellular stress, and immunogenic HMBG1 expression. Furthermore, activation of RIPK3-dependent necroptosis in A549 xenograft and orthotopic LLC1 tumor mouse models revealed potent antigen cross-presentation between DCs and $CD4^+$ and $CD8^+$ T cells.[22]

Here, we report the oncolytic efficacy of rVSV-NDV in the human non-small cell lung cancer (NSCLC) cell lines, A549 and H1437. Although rVSV- and rVSV-NDV-mediated oncolysis displayed cell line-specific kinetic and morphological differences, we observed a less clear dependency of fusogenicity in determining the resultant cell death pathways. However, genetic manipulation in the respective death pathways indicated that OV-mediated killing was not abrogated but proceeded in favor of an alternative pathway, substantiating the proposed complexity and parallel engagement of multiple death modalities during oncolysis. Interestingly, our results pinpoint a potential central role of caspase-3 during rVSV- and rVSV-NDV-mediated killing. Moreover, we demonstrate that oncolysates that undergo a syncytial form of cell death are more efficient in activating DCs, highlighting the superior immunological potential of fusogenic rVSV-NDV compared with fusion-incompetent rVSV.

RESULTS

Cell line- and virus-specific CPEs in human lung cancer cells

We sought to compare the viral growth kinetics and oncolytic susceptibility of two selected human NSCLC cell lines to fusogenic rVSV-NDV or fusion-incompetent rVSV. A549 and H1437 cells were infected at a multiplicity of infection (MOI) of 0.01, and viral replication and cell killing were examined over time. As previously reported, A549 cells were highly susceptible to rVSV infection,[23] demonstrated by rapid replication to high viral titers, peaking by 24 h post infection (hpi) (Figure 1A), and complete killing of the entire monolayer by 48 hpi (Figures 1B and 1C). In contrast, peak titers in H1437 cells were more than 1-log lower, and rVSV-mediated oncolysis was comparably slower (Figures 1D and 1E). rVSV-NDV also replicated to lower titers than rVSV in both human lung cancer cell lines (Figures 1A and 1D), but yet induced sufficient oncolysis measured by the lactate dehydrogenase (LDH) assay (Figures 1B and 1E). These data are consistent with previous observations in human HCC cells in which we consistently observe lower titers for rVSV-NDV compared with VSV, despite an equivalent level of cytotoxicity.[2] We attribute this observation to the efficiency of killing via cell-cell fusion that enables even few virions to efficiently kill many tumor cells.

In both cell lines, the CPEs of rVSV were characterized by single-cell rounding, despite cell line-specific differences, with a notably earlier infection of the entire monolayer in A549 cells, compared with slower viral spreading from distinct foci in H1437 cells (Figures 1C and 1F). Microscopy further revealed cell line-specific CPEs and syncytia morphologies after rVSV-NDV infection. rVSV-NDV-induced cell-cell fusions in A549 cells expanded to wide-spanning syncytia that shed off the monolayer as dead cells by 72 hpi, but left behind a subset of surrounding uninfected cells, even though the measured cytotoxicity might imply complete death (Figure 1B). In H1437 cells, rVSV-NDV spread in patches and formed more rounded syncytia of various sizes that burst more rapidly than in A549 cells, indicating slightly greater susceptibility of H1437 to rVSV-NDV (Figure 1E). Before loss of membrane integrity, enlargement of vacuole-like organelles and signs of membrane blebbing were apparent in both cell lines (Figures 1C and 1F).

To further distinguish the oncolysis process of the two OV variants and to quantify cell-to-cell fusion in NSCLC cells, a real-time assay based on a split NanoLuc luciferase was designed (Figure 1G). Plasmids encoding for the large (LgBiT) and high-affinity (HiBiT) subunits of the NanoLuc luciferase were each stably transfected into A549 or H1437 cells. Upon coculture and OV-induced cell-to-cell fusion, cytoplasmic mixing would complement the LgBiT and HiBiT luciferase subunits and, in the presence of the Endurazine live-cell pro-substrate, generate luminescence in an amount proportional to the extent of cell-to-cell fusion. When quantifying rVSV- or rVSV-NDV-induced cell-cell fusion and plotting the fold increase in fusion relative to uninfected cells, we confirmed that only rVSV-NDV attained high degrees of fusogenicity in a pattern that matched the observed syncytia formation in the respective cell line, while fusion-incompetent rVSV did not yield any luminescence signal (Figures 1H and 1I).

Dynamic engagement of intrinsic and extrinsic apoptosis during oncolysis independent of viral fusogenicity

With the observed disparities between fusogenic and non-fusogenic oncolysis and unique infection and fusion kinetics in NSCLC cells, we next aimed to decipher the underlying cell death mechanism associated with syncytia formation by investigating the involvement of apoptosis and necroptosis. We first monitored the potential of

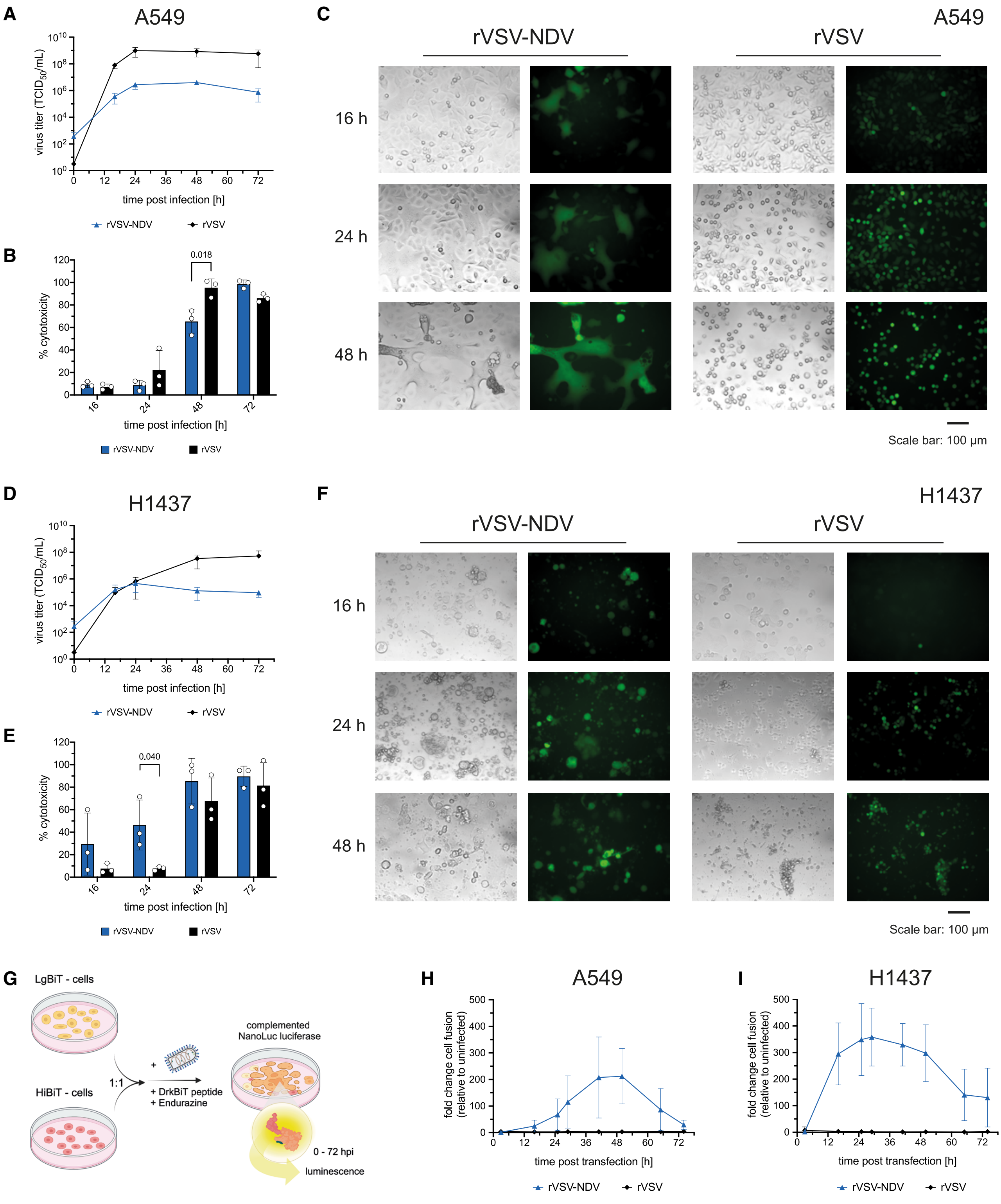

Figure 1. A549 and H1437 cell susceptibility to rVSV-NDV and rVSV infection

A549 and H1437 cells were infected at an MOI of 0.01 with rVSV-NDV-GFP or rVSV-GFP, and viral replication, oncolytic morphology, cytotoxicity, and fusogenicity were monitored over time. Viral replication in (A) A549 and (D) H1437 cells was monitored over time by $TCID_{50}$ assay from infectious supernatants. Virus-induced cytotoxicity

(legend continued on next page)

rVSV and rVSV-NDV to induce mitochondrial outer membrane permeabilization (MOMP) and activation of intrinsic apoptosis via caspase-9. Using carbonyl cyanide-*p*-trifluoromethoxyphenylhydrazone (FCCP) as an uncoupling agent, we confirmed that A549 and H1437 cells were capable of undergoing dose-dependent MOMP (Figure S1). Choosing a range of MOIs to guarantee all stages from early syncytia development to complete death, we detected an MOI-dependent reduction in tetramethylrhodamine (TMRE) fluorescence, indicative of MOMP, in both cell lines (Figures 2A and 2G). Consistent with the greater susceptibility of H1437 cells to rVSV-NDV oncolysis, we observed a lower MOI threshold to induce MOMP in H1437 than in A549 cells. These trends were inverted for non-fusogenic rVSV.

We next performed OV infections at an MOI of 1 and performed western blot analyses at early (8 hpi), intermediate (16 hpi), and late (24 hpi) stages of syncytia formation or rVSV-mediated CPEs. While staurosporine, as well as tumor necrosis factor (TNF)-α and Smac mimetic (TS), could not efficiently trigger initiator caspase-8 or -9 activity in A549 cells, moderate downstream caspase-3 activity was detectable (Figures 2B–2F). In contrast, H1437 cells responded to these apoptosis inducer controls with early initiator caspase-8 and -9 activation, followed by effector caspase-3 cleavage between 8 and 24 hpi (Figures 2H–2L). Unfortunately, as previously reported,[24] the effects of TSZ on dampening caspase activities were only minor in both cell lines. Although effects in A549 cells were masked by unexpectedly high baseline caspase-9 cleavage in uninfected cells, western blot trends nevertheless indicate an underlying caspase-9-mediated apoptosis over the course of infection with either virus (Figure 2B). While rVSV caused moderate and steady caspase-9 activation in H1437 cells, a strong peak of caspase-9 cleavage was detectable from rVSV-NDV-infected H1437 cells at an intermediate stage of syncytia formation (16 hpi) (Figure 2H), implying that a fusogenic death activates elements of intrinsic apoptosis.

To further delineate the involvement of apoptosis, we monitored activation and cleavage of initiator caspase-8, as well as downstream effector caspase-3 by western blot and enzymatic activity assays. In A549 cells, neither OV variant induced noteworthy levels of caspase-8 cleavage activity (Figure 2C), and only weak bands of the larger cleavage fragment (42 kDa), but not the small 18-kDa caspase-8 fragment (not shown), were detectable by western blot (Figure 2D). Thus, similar to the control treatments, staurosporine or TS, neither the involvement of initiator caspases-8 nor -9 during OV-mediated killing could be conclusively demonstrated in A549 cells. However, at time points where rVSV-NDV infection leads to observable syncytia (24 hpi) and obvious cell death (36–72 hpi), distinct enzymatic activity and cleavage of the effector caspases-3 and -7 were detectable (Figures 2E and 2F). Accumulating, but only moderate, caspase-3/7 activity was triggered by rVSV infection in A549 cells until the time of complete death at 48 hpi (Figure 2E). In contrast, enzymatic activity data from H1437 cells indicated a consecutive involvement of caspase-8, followed by caspase-3/7 activity during an rVSV- and rVSV-NDV-mediated death (Figures 2I and 2K), which is also partly reflected in early caspase-8 and caspase-3 protein cleavage shown by western blotting (Figures 2J and 2L). Even though results lack statistical significance, and the exact kinetic sequence of initiator and effector caspase activation could not be revealed with these experiments, we infer from the trends that both OVs dynamically engaged caspase-mediated apoptosis to promote oncolysis, albeit with cell line-specific differences and independent of viral fusogenicity.

To qualitatively investigate caspase-3 cleavage in the context of fusogenic and nonfusogenic OV infection, we performed immunofluorescent staining of cleaved caspase-3 in rVSV-GFP- or rVSV-NDV-GFP-infected A549 or H1437 in comparison with uninfected cells. Consistent with western blot and enzymatic activity data, only weak and sporadic amounts of cleaved caspase-3 could be detected in A549 cells after infection with either virus at 24 h (Figure S2A) or 48 h (Figure S2B), which coincided with areas of infection as determined by GFP expression. In H1437 cells, where infections were more advanced by 24 h, a notable increase in the amount and intensity of cleaved caspase-3 was detected in response to both viruses (Figure S3). Interestingly, in rVSV-NDV-GFP-infected cells, cleaved caspase-3 seemed to be enriched in smaller syncytium, while more expansive syncytium demonstrated a more diffuse staining. Because extensive cell death had already occurred by 24 h in H1437 cells, cleaved caspase-3 staining was not performed at a later time point.

Viral oncolysis coincides with downstream apoptotic effector functions independent of fusogenicity

We further characterized caspase-mediated apoptotic effector functions by investigating (poly-ADP)-ribose polymerase (PARP) cleavage, the early apoptosis exposure of phosphatidylserine (PS) on the plasma membrane, as well as signs of secondary necrosis. Western blot analysis indicated a stepwise increase in PARP cleavage in A549 or H1437 cells infected with rVSV, whereas a syncytial death mediated by rVSV-NDV only caused minimal PARP cleavage in A549 cells but a high magnitude of cleavage in H1437 cells (Figures 3A and 3D). Overall, the time of measurable peaks in

measured from infectious supernatant of (B) A549 and (E) H1437 cells was measured using the Cytotox96 Non-radioactive cytotoxicity assay (LDH release). Values are expressed as percent cytotoxicity relative to a maximum release (100% cytotoxicity) control. Representative fluorescent and brightfield microscopy images of (C) A549 and (F) H1437 cells infected with rVSV-GFP or rVSV-NDV-GFP monitored at 16–48 hpi from $n = 3$ independent biological replicates are shown. Images have been adjusted to similar contrast and brightness. (G) Schematic overview of the developed luminescence assay to monitor OV-mediated cell-cell fusion in real time (image created with BioRender). Real-time measurement of OV-mediated cell-cell fusion in (H) A549 and (I) H1437 cells, expressed as the fold-change relative to uninfected cells. Mean values ± SD of $n = 3$ (LDH and $TCID_{50}$ assay) or $n = 4$ (fusion assay) independent experiments are shown. Statistical significance in (B) and (E) was determined by unpaired two-tailed Student's t test, and p values are indicated.

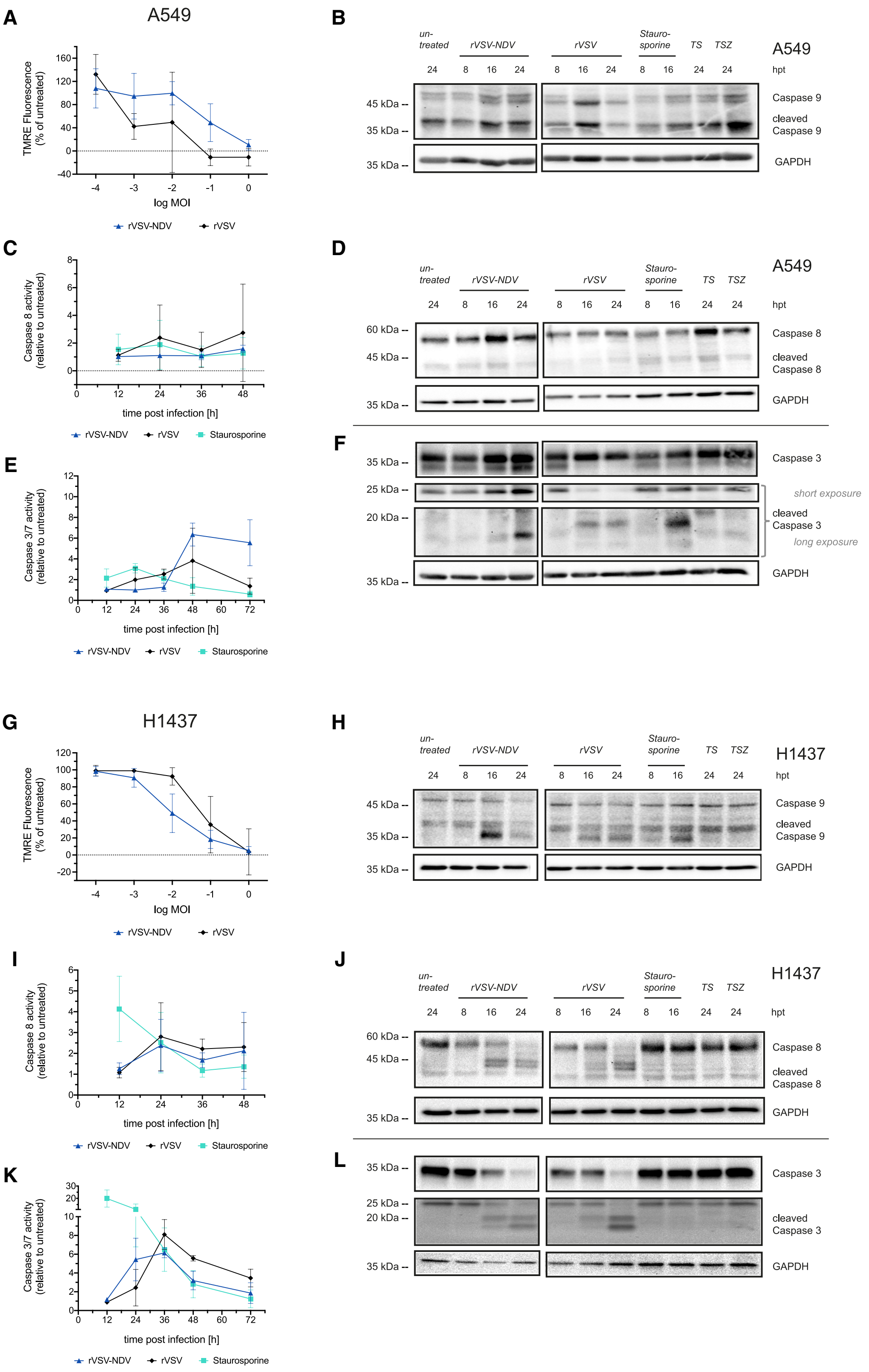

(legend on next page)

PARP cleavage seemed to correlate with syncytia formation and onset of their death. We further evaluated apoptotic effector functions by using the Realtime-Glo Annexin V Apoptosis and Necrosis assay. Even though the results were subject to high variation, results from rVSV-infected A549 cells indicate moderate PS exposure throughout infection, which seemed to be followed by secondary necrosis (Figures 3B and 3C). In contrast, H1437 cells responded to rVSV infection with a late peak of PS exposure but only low levels of secondary necrosis (Figures 3E and 3F). Interestingly, in response to rVSV-NDV infection, both cell lines displayed PS exposure and secondary necrosis profiles that correspond well with the previously described LDH release kinetics and microscopically observed syncytial stages (Figure 1). A slower progressing syncytial death in A549 cells triggered late exposure of PS on syncytial membranes, which eventually collapsed by necrosis (Figures 3B and 3C). Similarly, but with a faster kinetic, H1437 cells infected with rVSV-NDV exposed PS on syncytial membranes and increasingly disintegrated fused cells by secondary necrosis (Figures 3E and 3F). Consequently, oncolysis by fusogenic rVSV-NDV or parental rVSV both coincided with a dynamic caspase-mediated apoptotic death program in NSCLC cells, which likely culminated in a necrotic destruction of infected cells late in infection. Unfortunately, the commercial assay used here was associated with a relatively high degree of inter-experimental variation in signal intensity, which resulted in broad error bars. However, since the overall trends were conserved across experimental datasets and reflect the respective susceptibility of the two cell lines to the OVs, we believe that the overall kinetics of PS exposure and necrosis hold true.

Evidence of necroptotic signaling is observed in response to VSV- or VSV-NDV-mediated oncolysis

To further determine the necrotic nature of a fusogenic and non-fusogenic OV-mediated death, we analyzed the expression and phosphorylation of the necroptosis mediators, RIPK1, RIPK3, and MLKL, over the time course of infection by western blot analysis. Of note, we repeatedly detected a bright band of a potential additional RIPK3 isoform in rVSV-infected cells that accumulated at 16 and 24 hpi above the reported 57 kDa RIPK3 band (Figures 4A and 4C). It is plausible that this represents a RIPK3 isoform and accounts for a stronger RIPK3 involvement in rVSV- rather than in rVSV-NDV-mediated death; however, since such an isoform has not been previously reported (to our knowledge), we did not consider this band in our analysis.

Although the calculated fold changes for RIPK1 and RIPK3 phosphorylation were rather low and subject to high inter-experimental variation over the time course of infection, trends point toward an increased expression and phosphorylation of MLKL in rVSV-infected A549 and H1437 cells 16–24 hpi (Figure 4). This implied that *p*-MLKL-derived membrane pores may eventually control necrosis of rVSV-infected cells. Moreover, rVSV-NDV similarly triggered a small but gradual increase in phosphorylation of MLKL from 16 to 24 hpi in both cell lines. This presumably leads to syncytial necroptosis, although, except for a peak at 24 hpi in H1437 cells, preceding RIPK1 phosphorylation was hardly detected, and the fold changes of RIPK3 phosphorylation were negligible in both of the cell lines. Nevertheless, we conclude that both OV variants exhibited some necroptotic signaling, albeit in an inconclusive pattern. Given the lack of statistical significance in our data, we further attempted to delineate a central underlying death mediator by genetically manipulating the respective cell death pathways.

Genetic deletion of mutual death mediators alters the oncolytic program

To further interrogate the role of cell death pathways on the fate of oncolytic viral activity, we genetically manipulated key apoptotic or necroptotic regulators and monitored the viability, oncolytic phenotypes, and viral replication over the course of OV infection. First, single-cell clone CRISPR-Cas9 knockouts of mutual death pathway regulators, caspase-8, RIPK1, or RIPK3, and two polyclonal non-target controls (NTC1 and NTC2) were generated in A549 and H1437 cells. Successful gene deletion was validated by western blot and PCR over the CRISPR-targeted exons (Figure S4). In a time course infection experiment, we examined changes in oncolysis by plotting log2-fold changes in viability, relative to the average of both NTCs (Figures 5A and 5C) and followed the infection by fluorescent microscopy (Figures 5B and 5D). Due to cell-line-specific viral kinetics, the respective peak time point of observable syncytia formation (48 hpi for A549 and 36 hpi for H1437) are compared.

The absence of effector caspase-8 visually promoted rVSV-NDV infection in both cell lines when observed microscopically, but a greater degree of syncytial lysis was only measurable in H1437 caspase-8$^{-/-}$ cells. With respect to the previously observed caspase activity data (Figure 2), we assume that these trends could point to a switch to necroptosis or upregulated intrinsic apoptosis. Such pathway flexibility during syncytial oncolysis appeared to be

Figure 2. Apoptosis characterization during infection with oncolytic rVSV or rVSV-NDV in A549 and H1437 cells
Permeabilization of the outer mitochondrial membrane (MOMP) was measured in (A) A549 and (G) H1437 cells after 36 h of rVSV or rVSV-NDV infection at a series of MOIs using the mitochondrial fluorescent dye TMRE, and data are expressed as percent fluorescence reduction relative to untreated cells. Full-length and cleaved caspase-9 protein expression at 8, 16, and 24 h after rVSV or rVSV-NDV infection at MOI 1 in (B) A549 or (H) H1437 cells were measured by western blot analysis. Enzymatic activity of caspase-8 was measured after OV infection at MOI 1 using the Caspase-8 Glo activity assay in (C) A549 or (I) H1437 cells. Data are plotted as the fold-change relative to untreated cells. Full-length and cleaved caspase-8 protein expression at 8, 16, and 24 h after rVSV or rVSV-NDV infection at MOI 1 in (D) A549 or (J) H1437 cells were analyzed by western blot. Enzymatic activity of caspase-3/7 measured after OV infection at MOI 1 using the Caspase-3/7 Glo activity assay in (E) A549 or (K) H1437 cells. Data are plotted as the fold-change relative to untreated cells. Full-length and cleaved caspase-3 protein expression at 8, 16, and 24 h after rVSV or rVSV-NDV infection at MOI 1 in (F) A549 or (L) H1437 cells were analyzed by western blot analysis. Representative western blots of $n = 3$ independent biological replicates are shown. MOMP and caspase activity data are plotted as mean (± SD) values of $n = 3$ independent biological replicates. Western blots were cropped at the solid vertical lines.

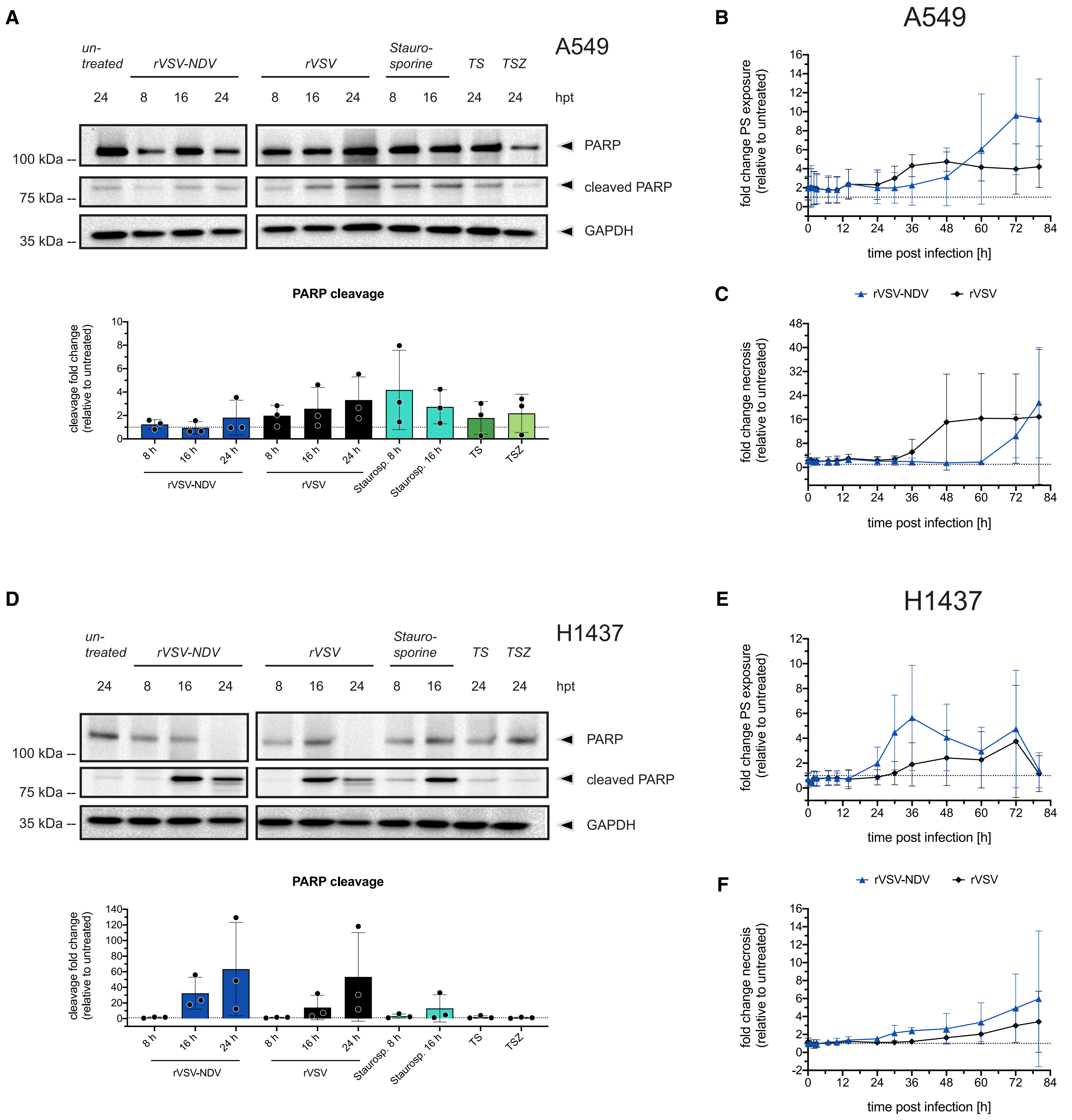

Figure 3. Apoptosis effector functions in rVSV- or rVSV-NDV-infected A549 and H1437 cells

Full-length and cleaved PARP protein expression were measured at 8, 16, and 24 h after rVSV or rVSV-NDV infection at MOI 1 in (A) A549 or (D) H1437 cells by western blot analysis and quantified relative to GAPDH expression and expressed as the fold-change relative to untreated cells. Representative western blots and quantifications of $n = 3$ independent biological replicates are shown. Western blots were cropped at the solid vertical lines, and arrowheads on the right indicate the quantified protein bands. Real-time monitoring of virus-induced PS exposure (B and E) and secondary necrosis (C and F) after rVSV or rVSV-NDV infection at MOI 0.01 was performed in A549 or H1437 cells using the Realtime-Glo Annexin V Apoptosis and Necrosis assay. The fold changes of luminescence (PS exposure) or fluorescence (secondary necrosis) relative to untreated cells are plotted. Data are represented as mean (± SD) values of $n = 3$ independent biological replicates.

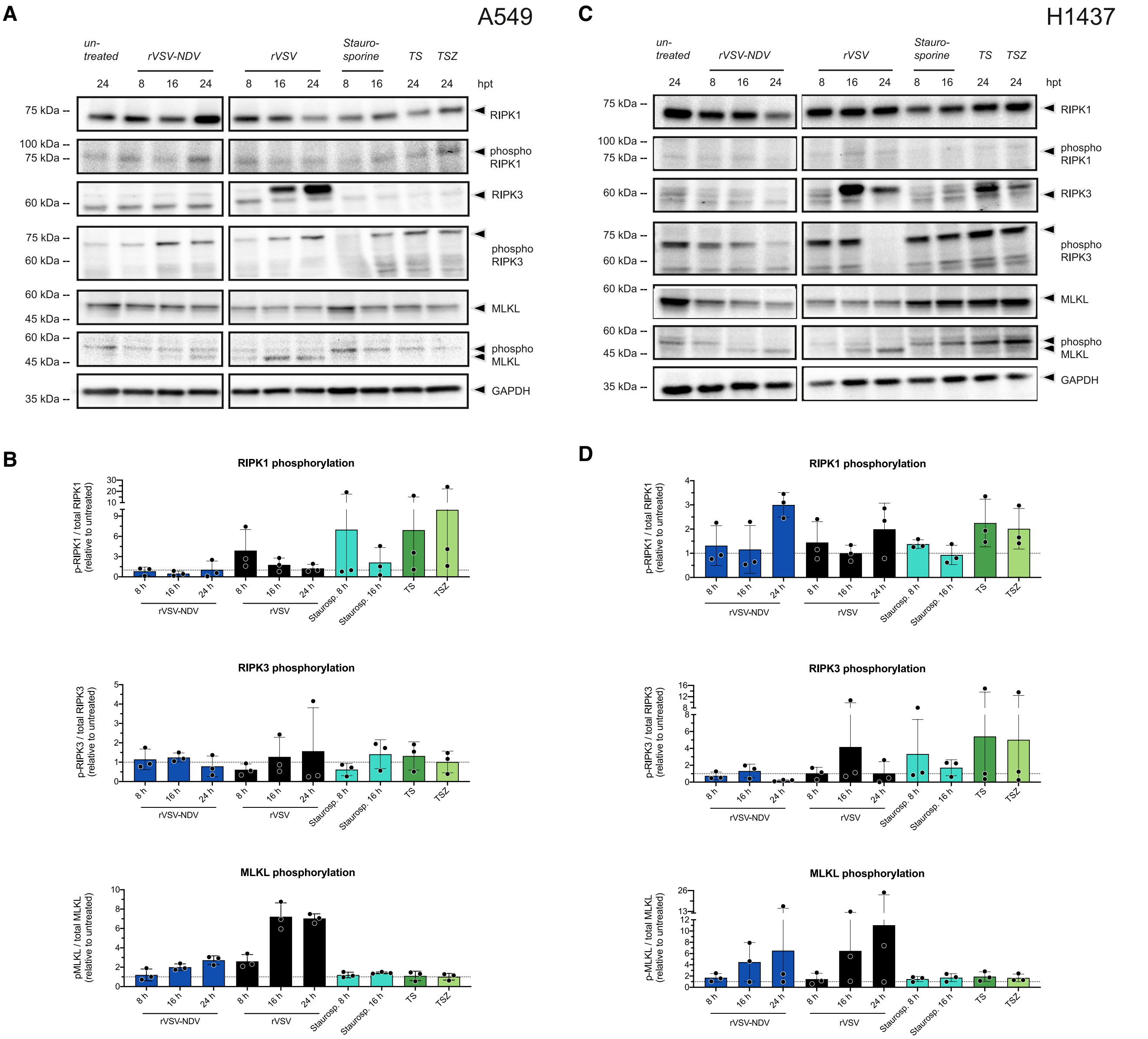

Figure 4. Necroptosis characterization during infection with oncolytic rVSV or rVSV-NDV in A549 and H1437 cells

A549 and H1437 cells were infected with either rVSV or rVSV-NDV at an MOI of 1. Lysates were collected at 8, 16, and 24 hpi and analyzed by western blot for the expression and phosphorylation of necrosome components RIPK1, RIPK3, or MLKL. Representative western blots of OV-infected (A) A549 and (C) H1437 were cropped at the solid vertical lines, and arrowheads on the right indicate the quantified protein bands. For simplicity, only one representative GAPDH blot is shown. Corresponding quantification of RIPK1, RIPK3, or MLKL phosphorylation was derived from the protein expression of unphosphorylated and phosphorylated protein relative to the respective GAPDH expression and is visualized in (B and D) as the phosphorylation fold-change relative to untreated cells. Representative blots of n = 3 independent biological replicates are plotted and mean (± SD) values of n = 3 independent biological replicates are shown.

generally greater in H1437 cells than in A549 cells. In H1437 cells with a knockout of upstream necroptosis kinase, RIPK1, or RIPK3, we observed slightly diminished cell-cell fusion and maintained viability compared with NTCs, indicating that rVSV-NDV-mediated syncytial death seemed to have some level of dependence on RIP kinase activity. In the respective A549 knockout cells, attenuated syncytial effects were similarly observed by microscopic analysis, but the quantitative viability measurements were only marginally influenced by the deletion of RIPK1 or RIPK3. Taken together, we speculate that, depending on the cellular background, fusogenic rVSV-NDV can dynamically alternate to presumably RIPK1-, RIPK3-dependent necroptotic syncytia destruction when caspase-8 is absent. On the

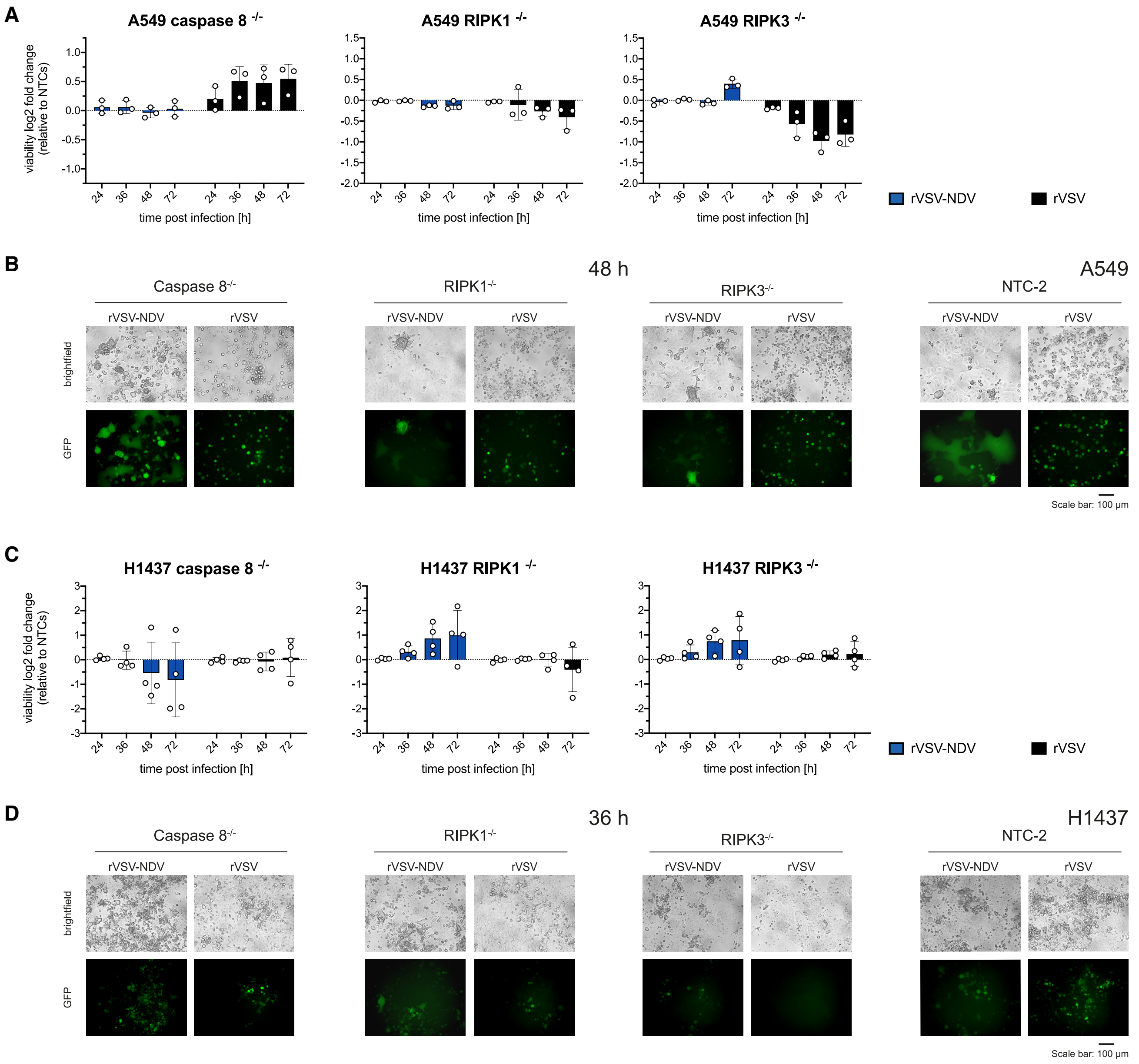

Figure 5. Impact of genetic deletion of upstream cell death mediators caspase-8, RIPK1, or RIPK3 on rVSV- or rVSV-NDV-mediated oncolysis in A549 and H1437 cells

Gene knockouts for caspase-8, RIPK1, or RIPK3 and two NTCs were generated using CRISPR-Cas9 technology. Validated single-cell knockout clones of (A and B) A549 and (C and D) H1437 cells were infected with GFP-expressing rVSV or rVSV-NDV at an MOI of 0.01, and changes in cell viability were measured by CellTiter-Glo assay in a time course experiment. (A and C) Viability data were normalized to uninfected cells and expressed as log2-fold changes to the average of NTC1 and NTC2. Mean values (± SD) of n = 3 (A549) or n = 4 (H1437) independent biological replicates are plotted. Representative fluorescent and brightfield microscopy images (n = 3 independent biological replicates) of OV-infected (B) A549 or (D) H1437 knockout cells are shown. Microscopy images have been adjusted to similar contrast and brightness.

contrary, data from fusion-incompetent rVSV revealed boosted oncolysis in A549 RIPK1$^{-/-}$ and RIPK3$^{-/-}$ cells but impaired death in absence of caspase-8 at almost all time points, which suggests a considerable dependency of rVSV on caspase-8-mediated extrinsic apoptosis rather than on necroptosis. However, contrasting results were obtained in H1437 knockout cells, where none of the gene knockouts had a substantial effect on VSV-mediated cytotoxicity. Based on these findings, we conclude that the modulation of cell death pathways has an impact on OV activity; however, these effects are both cell line and virus specific.

Caspase-3 as a central player during rVSV-NDV-mediated syncytial cell death

Having seen prominent shifts in syncytial morphology and death progression in the absence of mutual, upstream cell death mediators, caspase-8, RIPK1, or RIPK3, we next aimed to identify which of the downstream cell death effectors, caspase-3 or MLKL, plays a crucial role in fusogenic or non-fusogenic death. In a similar manner, we generated single-cell clone CRISPR-Cas9 knockouts of caspase-3, or MLKL in A549 and H1437 cells (validated in Figure S4) and carried out a time course infection experiment compared with NTCs. Again, due to cell-line specific viral kinetic, the respective peak time point of observable syncytia formation (48 hpi for A549 and 36 hpi for H1437) are presented.

While rVSV-NDV infection in caspase-3-deleted A549 cells had no clear effect on measured cell viability, oncolysis of H1437 caspase-3$^{-/-}$ cells was markedly diminished compared with NTCs throughout infection with the fusogenic OV (Figures 6A and 6C). Notably, a lack of caspase-3 caused a longer persisting and distinctive syncytial phenotype in A549 and, especially in H1437 cells (Figures 6B and 6D). Here, it seems that the effect of caspase-3 on sustaining syncytia viability is more ubiquitous across cell lines and species. Interestingly, we further validated this phenotype in B16-OVA cells as a representative murine tumor cell line of a different tumor indication. B16-OVA cells have been previously characterized as highly susceptible to rVSV-NDV infection and oncolysis *in vitro* and *in vivo*.[3,25] Using B16-OVA cells bearing a caspase-3 deletion, we similarly observed enhanced syncytia development and delayed cell death as determined by LDH assay, overall supporting the idea of a caspase-3-controlled syncytial destruction (Figure S5).

We initially speculated a central role of MLKL during syncytial death. However, in H1437, we observed enhanced oncolysis, indicating that rVSV-NDV-mediated oncolysis is not necessarily dependent on MLKL, and the virus can seemingly exploit an alternative non-necroptotic pathway in the absence of MLKL (Figure 6D). Similar results were achieved in A549 (Figures 6A and 6B), although the enhanced cytotoxicity in MLKL knockouts is better observed microscopically than quantitatively. Interestingly, in addition to an enhancement in oncolysis in both cell lines, we also observed an obvious change in the morphology of the cells upon rVSV-NDV infection (Figures 6B and 6D), shifting from a syncytial death to a more classically rounded CPE, which was also observed in B16-OVA cells (Figure S3B).

In agreement with the literature describing a strong reliance of rVSV on caspase-mediated apoptosis,[13] and further demonstrated in caspase-8-deleted A549 (Figure 5), knockout of effector caspase-3 similarly increased cellular viability compared with NTCs in both cell lines (Figure 6). Surprisingly, this effect was accompanied by a marked change in cell morphology after VSV infection. Especially prominent in A549, VSV-infected cells are substantially enlarged and rounded. This is consistent with a delayed burst, which allowed for prolonged intracellular virus production, leading to the observed swelling. In contrast, genetic deletion of the necroptotic regulator MLKL in A549 and H1437 cells considerably boosted rVSV-mediated oncolysis at almost all time points (Figure 6), substantiating the idea of a dominant apoptosis-dependent cell death program. Of note, rVSV infection of B16-OVA cells with either a caspase-3 or MLKL knockout neither led to a markedly altered cellular morphology nor significantly delayed or promoted cytotoxicity (Figure S5).

In addition to quantifying cytotoxicity, we also questioned whether altered oncolysis in the applied A549 or H1437 CRISPR knockout cells impacted changes in viral replication of the fusogenic or non-fusogenic OV variant. However, measurement of rVSV and rVSV-NDV titers in the supernatants revealed no significant differences between any of the gene knockouts compared with the NTCs (Figure S6). This indicates that both virus variants maintained their replication independent of an altered apoptotic or necroptotic death signaling and suggest that additional death pathways play a role during the OV life cycle. However, we cannot rule out that either cell death pathway was completely disrupted by the knockout of a single gene and, therefore, we cannot draw definitive conclusions on the role of either pathway in modulating the OV life cycle.

We further intervened in the respective death pathway by infecting wild-type, caspase-3$^{-/-}$, or MLKL$^{-/-}$ cells in combination with pharmacological pan-caspase inhibition (zVAD), inhibition of RIPK1 (Nec-1), RIPK3 (GSK-872), or MLKL (NSA). However, none of the drug treatments yielded quantifiable alterations in syncytial oncolysis compared with untreated wild-type or knockout cells. Although, the above presented data indicate phosphorylation of MLKL during oncolysis (Figure 4), neither genetic inhibition of MLKL nor additional pharmacological inhibition of RIP1, RIPK3, or MLKL could prevent viral oncolysis, suggesting parallel involvement of alternative, non-necroptotic death pathways. Interestingly, zVAD could only visually mimic the phenotypic effects of a slowed down syncytial death progression seen in caspase-3 knockout cells (data not shown).

All in all, we were able to delineate a central role of caspase-3 during rVSV- and particularly rVSV-NDV-mediated oncolysis that warrants further mechanistic investigation, especially with respect to a potential parallel engagement of multiple death modalities during oncolysis.

Syncytial death by rVSV-NDV elicits superior *in vitro* immunogenicity

Previous work in our group has demonstrated substantial immunogenicity of fusogenic rVSV-NDV in human HCC and murine melanoma models.[2–4] Here, we further explored the immunogenicity of the fusogenic cell death mediated by rVSV-NDV in two human NSCLC cell lines by characterizing the exposure and release of hallmark danger-associated molecular patterns (DAMPs) and by quantifying the activation of co-cultured human dendritic cells (DCs) *in vitro*, as a functional readout. We first measured the level of

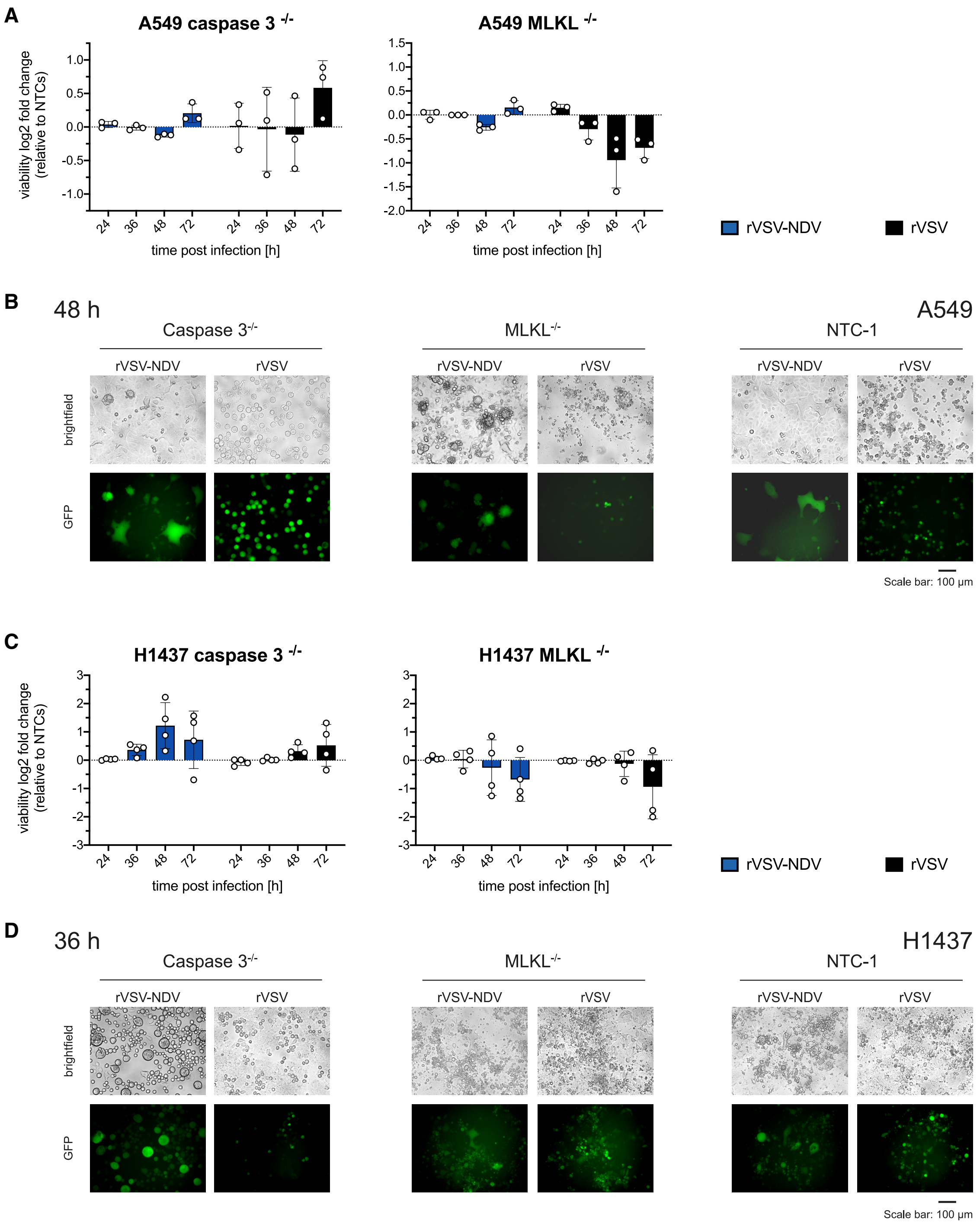

Figure 6. Impact of genetic deletion of downstream apoptosis or necroptosis effectors on rVSV- or rVSV-NDV-mediated oncolysis in A549 and H1437 cells
Gene knockouts for caspase-3 or MLKL and two NTCs were generated using CRISPR-Cas9 technology. Validated single-cell knockout clones of (A and B) A549 and (C and D) H1437 cells were infected with GFP-expressing rVSV or rVSV-NDV at an MOI of 0.01, and changes in cell viability were measured by CellTiter-Glo assay in a time course experiment. (A and C) Viability data were normalized to uninfected cells and expressed as log2-fold changes to the average of NTC1 and NTC2. Mean values (± SD) of $n = 3$ (A549) or $n = 4$ (H1437) independent biological replicates are plotted. Representative fluorescent and brightfield microscopy images ($n = 3$ independent biological replicates) of OV-infected (B) A549 or (D) H1437 knockout cells are shown. Microscopy images have been adjusted to similar contrast and brightness.

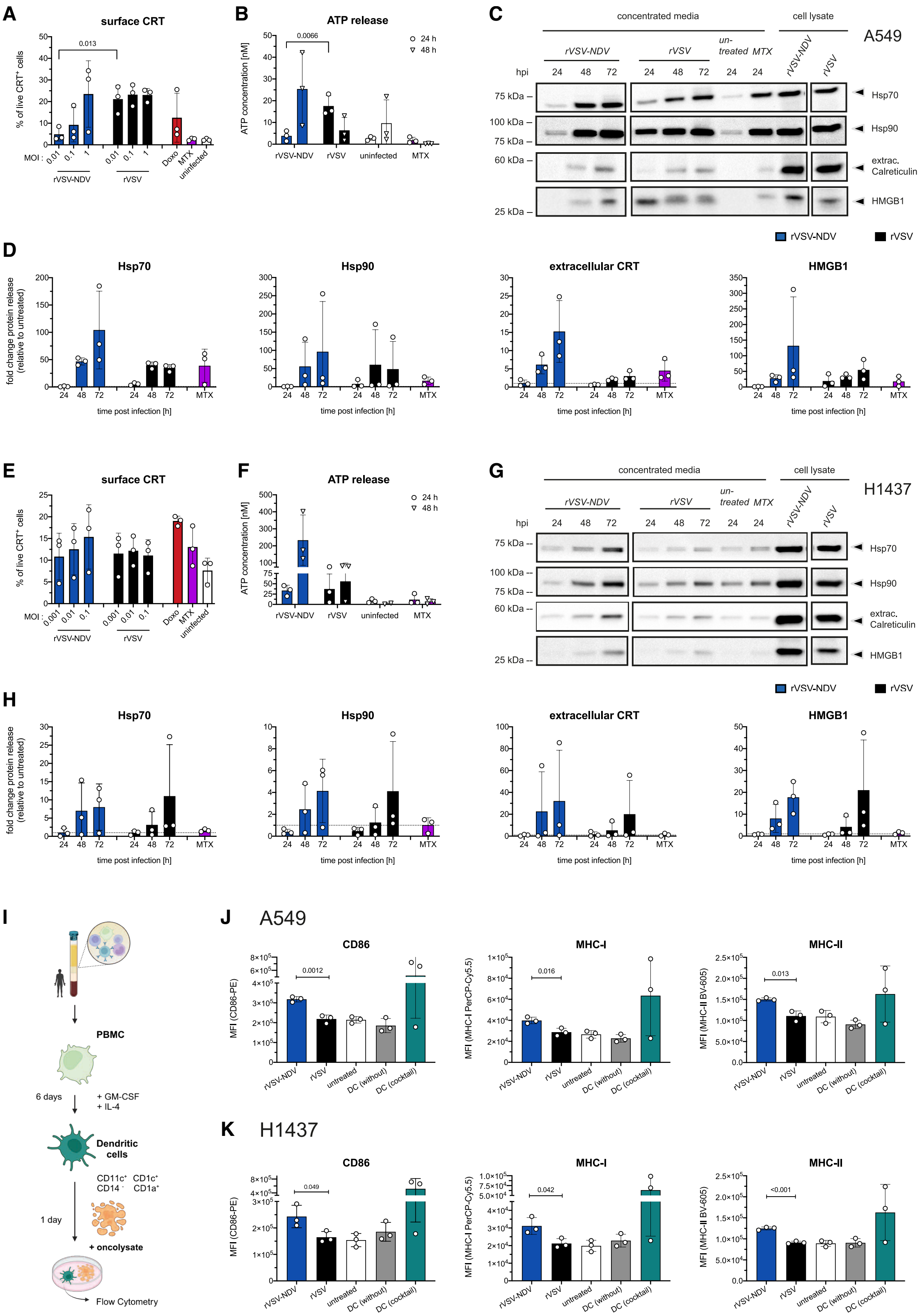

(legend on next page)

surface exposed calreticulin (CRT) at 24 hpi as an early apoptotic "eat me" signal[26] on infected living cells using flow cytometry. As expected, doxorubicin and, to a lesser extent, MTX induced exposure of CRT on the surface of A549 and H1437 cells, confirming that cells are responsive to ICD stimuli. Interestingly, both viruses induced the presence of surface exposed CRT in both cell lines, which appeared to be MOI-dependent for fusogenic rVSV-NDV in A549 cells (Figures 7A and 7E).

We next quantified the level of released ATP in OV-infected or MTX-treated supernatants. In line with the relative susceptibilities of the two cell lines, both OVs caused greater ATP release than the ICD inducer, MTX, with peaks for rVSV-NDV reaching substantially higher levels than rVSV at 48 hpi, which was a magnitude higher in H1437 than in A549 cells (Figures 7B and 7F). To complement the soluble ICD marker characterization, western blot time course analysis of concentrated supernatants was performed. Quantification of the fold-protein release revealed accumulation of heat shock protein (HSP)70, HSP90, HMGB1, and CRT in supernatants of rVSV- and rVSV-NDV-infected A549 and H1437 cells over the entire course of infection and to much greater degrees than MTX-treated control cells (Figures 7C, 7D, 7G, and 7H). Trends in A549 cells indicated slightly higher CRT and HSP70 release from cells infected with the fusogenic variant, although protein quantifications did not yield statistically significant fold differences due to variations between the individual western blots. Despite high inter-experimental variations, and the fact that western blot data are only semi-quantitative, we see indications that both OV variants induced the expression and exposure of immunogenic DAMPs in infected NSCLC cells.

To determine the role of caspase-3 and MLKL in mediating VSV-NDV-associated ICD, ATP release and cell membrane-associated CRTs were analyzed as representative ICD markers in caspase-3 and MLKL knockouts of A549 and H1437 in comparison with their wild-type counterparts or NTCs in the context of infection with increasing MOIs (MOIs of 0.01–1) of virus. Interestingly, at 24 hpi, NTCs of A549 cells released notably more ATP than wild-type cells in response to virus infection, which was significantly higher than that released by both knockouts at all MOIs (Figure S7A). At 48 h, ATP release was generally higher, and caspase-3 and MLKL knockouts released significantly less compared with either wild-type cells or NTCs when infected with an MOI of 0.01 (Figure S7B). In the H1437 background, all variants released much greater amounts of ATP overall, and the caspase-3 and MLKL knockouts released significantly less than the wild-type cells or NTCs when infected at an MOI of 0.1 at 24 h (Figure S7A), while no differences could be observed at 48 h (Figure S7B), presumably because the cells were already all dead by that time point. In contrast, no differences in surface CRTs could be observed among knockouts of either cell background after rVSV-NDV infection, although there were slight trends toward lower percentages of CRT expression on the knockout cells (Figure S7C).

In addition to DAMP release, the immunogenic nature of viral oncolysis strongly relies on the recruitment and stimulation of antigen-presenting cells, such as DCs, which sense danger signals, cross-present neoantigens to naive cytotoxic and helper T cells, and eventually spark antigen-targeted, pro-inflammatory anti-tumor immune responses.[27] DC activation has been well characterized as a crucial readout of ICD.[28–30] We thus questioned whether syncytial and non-syncytial death suffice to potently stimulate human DCs *in vitro* as a functional readout of the immunogenicity of the respective mechanisms of cell death. To test this, we adapted a standard DC pulsing assay, which we had recently described in the murine system.[4,25] The assay comprises human peripheral blood mononuclear cell (PBMC)-derived naive DCs in co-culture with UV-inactivated oncolysates from infected tumor cells, and it measures upregulation of DC differentiation and co-stimulatory markers. Oncolysates were UV inactivated to avoid direct stimulation of DCs through infection with live virus. Although we cannot completely rule out that UV-inactivated virus did not lead to any direct activation of DCs in this context, previous (unpublished) studies in our lab indicate that exposure of DCs to the levels of UV-inactivated virus used here lead to minimal effects on DC activation markers. Similarly, direct infection with equivalent amounts of live virus also have negligible effects on human DCs in our hands. Human PBMCs were differentiated to a cDC2 subtype (confirmed by flow cytometry as $CD14^{-}$, $CD123^{-}$,

Figure 7. Characterization of ICD in rVSV- or rVSV-NDV-infected A549 or H1437 cells
A549 and H1437 cells were infected at an MOI of 0.01 or a range of MOIs (in A and E) with either rVSV or rVSV-NDV, or treated with MTX or doxorubicin as ICD inducer control. Flow cytometry-based quantification of CRT cell surface exposure in OV-infected A549 (A) and H1437 (D) cells at 24 hpi was calculated as percent CRT-positive cells among live cells and represented as mean (± SD) values of $n = 3$ independent biological replicates. Statistical significance was tested using one-way ANOVA with Tukey's multiple comparisons test. (B and F) Extracellular ATP quantified from supernatants of OV-infected A549 and H1437 cells at 24 and 48 hpi using an ATP Bioluminescent Assay kit. ATP concentrations in [nM] were calculated from a log-log standard curve generated with the supplied ATP standard (standard curve range 10 μM to 0.1 nM ATP). Plotted are mean (± SD) values of $n = 3$ independent biological replicates. Western blot detection of released HSP70, HSP90, HMGB1, and CRT from supernatant of OV-infected A549 (C) and H1437 (G) cells at 24, 48, and 72 hpi was performed. One representative blot of the time course infection experiments is shown, and RIPA whole-cell lysates were included as control. Western blots were cropped at the solid vertical lines. Protein bands from OV-infected A549 (D) or H1437 (H) cells were quantified relative to a Ponceau-S total protein staining (in each lane, not shown) and are expressed as protein release fold-change relative to untreated cells as mean (± SD) values of $n = 3$ independent biological replicates. (I) Human cDC2s were isolated from PBMCs and subsequently co-cultured with oncolysates of A549 and H1437 cells to characterize human DC differentiation and activation (created in BioRender; Kortum, F. [2025] https://BioRender.com/x16f126). Flow cytometry-based quantification of DC co-stimulatory markers from co-cultures of cDC2 cells with A549 (J) or H1437 (K) oncolysates are expressed as mean fluorescence intensity (MFI) of CD86, MHC-I, and MHC-II, respectively. Cocktail-stimulated DCs were used as a positive control, and non-co-cultured DCs (without) were used as an additional control. Mean (± SD) values of $n = 3$ individual healthy human donors are shown. Statistical significance for all graphs was tested using one-way ANOVA with Tukey's multiple comparisons test, and p values are indicated.

CD11c$^+$, CD1c$^+$, MHC-II^{high}, and MHC-I^{high}) (Figure S5A), and co-cultured for 24 h with oncolysates from OV-infected A549 or H1437 cells (Figure 7I). Non-cocultured DCs treated with an activation cocktail were used as a positive control and to establish the gating workflow (Figure S5B) and yielded high mean fluorescent intensity values for CD86, MHC-I, and MHC-II. Comparing oncolysates from rVSV- and rVSV-NDV-infected tumor cells, we observed a significantly greater upregulation of MHC-I, MHC-II, and CD86 on DCs that had been co-cultured with oncolysates from syncytia-forming rVSV-NDV (Figures 7J and 7K). Interestingly, oncolysates from non-fusogenic rVSV did not lead to the activation of DCs above levels obtained from uninfected control lysates. It should be noted that inter-donor variability in *ex vivo* immune cell responses is a common challenge leading to a broader degree of error bars. Interestingly, trends among cells derived from individual donors are observable (i.e., some donors yield DCs that are more highly activatable than others), while the overall trends across donors were conserved. As the effects were apparent in both cell lines and did not differ significantly between female and male donors, we conclude a superior *in vitro* immunogenicity from fusogenic rVSV-NDV-derived oncolysates than from fusion-incompetent rVSV-derived oncolysates.

DISCUSSION

In 2002, Bateman and colleagues[5] described tumor cell syncytia as highly organized cellular structures that dismantle in an immunogenic fashion, highlighting a potential therapeutic benefit of syncytium-based tumor cell lysis to potently stimulate antigen-presenting immune cells. To date, much of the mechanistic complexity underlying syncytial cell death, however, has remained undeciphered. We have previously described a novel oncolytic hybrid virus, rVSV-NDV, that elicits unique CPEs via syncytia formation in a broad range of tumor cell lines, and potently stimulates anti-tumor immune responses *in vitro* and *in vivo*.[2–4,25] With the current work, we aimed to decipher an underlying cell death pathway of rVSV-NDV-mediated syncytial oncolysis and to identify a potential mechanistic link between fusogenicity and immunogenicity. To do so, we compared classical cytopathic cell rounding induced by the parental rVSV with the cytolytic effects of syncytia-forming rVSV-NDV and report novel results on the involvement of two well-characterized cell death pathways, namely, apoptosis and necroptosis. While we demonstrate that rVSV-NDV and rVSV efficiently infect and destroy human lung cancer cells, the respective roles of apoptosis and necroptosis in the progression of syncytia formation and cell death were inconclusive and highly cell line and context dependent. A dynamic engagement of an immunogenic type of apoptosis and necroptosis signaling was suggested, although mostly independently of fusion capacity and often not statistically significant. Nevertheless, our data hinted at a central role of caspase-3 in mediating syncytial disintegration and in the burst of rVSV-infected cells. Strikingly, we highlight the superior ability of the fusogenic rVSV-NDV vector to potently stimulate human DCs in an *ex vivo* co-culture system.

In this work, we focused on human lung cancer and selected two cell lines with differing susceptibilities to OV infection. H1437 cells were notably more susceptible to infection with rVSV-NDV than A549, which seems to be at least partially related to a reduction in viral RNA sensors (i.e., RIG-I and PKR) in H1437 cells, based on preliminary unpublished data. A549 cells have been previously described as partially functional in type I interferon (IFN) signaling, which could explain why rVSV-NDV infection was less efficient in this cell line. Since rVSV replication is reproducibly faster and more robust than rVSV-NDV across multiple cell lines, it is likely that the differences in IFN signaling in these two cell lines play less of a role in VSV replication, providing a possible explanation as to why the cell-specific differences in viral growth kinetics were more prominent for rVSV-NDV.

When comparing the susceptibility of A549 or H1437 cells with fusion-incompetent rVSV and fusogenic rVSV-NDV, we detected considerable morphologic and kinetic differences in oncolysis that were also reflected in the underlying molecular cell death signatures. A classical single-cell infection by rVSV caused rounding and shrinkage of infected lung tumor cells and was also mechanistically characterized as prototypic apoptotic death. Time course studies of rVSV infection pointed to a consecutive activation of intrinsic MOMP-caspase-9 signaling, as well as caspase-8-mediated extrinsic apoptosis, both leading to caspase-3/7 effector functions. This is in line with existing data and the known ability of rVSVs to activate and alter aspects of apoptosis signaling to maintain oncolysis.[12–14,31] The concept of the apoptosis dependency of rVSV was further supported by a substantial delay of oncolysis in caspase-3$^{-/-}$ cells. Interestingly, absence of caspase-3 induced enlargement of rounded rVSV-infected cells, similar to observations reported by Hobbs et al.,[32] which could be interpreted as a mechanistic switch to necroptotic cell swelling. Deletion of effector caspase-8 only delayed rVSV-mediated death in A549 cells, while H1437 cells maintained similar killing kinetics as NTC cells. We interpret this as poor flexibility of A549 cells to upregulate caspase-9-mediated intrinsic apoptosis or alternate to necroptosis (as further mentioned below). By western blot analysis, we established that rVSV also activated MLKL-mediated necroptotic signaling in both NSCLC cell lines, even without exogenous caspase-8 inhibition (e.g., by zVAD addition), which has so far only been sporadically reported.[33] However, the determination of which of the numerous molecular events that are required to cause the switch from apoptotic signaling to necroptosis, such as internalization of death receptor complex II, proper de-ubiquitination, and phosphorylation of RIP kinases, and caspase-8 inactivation by, for example, c-FLIP,[34] which was causative during rVSV-activated necroptosis in NSCLC cells, was not resolved in our work. Nevertheless, our data allow the presumption that the absence of necroptotic regulators, RIPK1, RIPK3, or MLKL, triggered rVSV to rapidly compensate the underlying cell death program and upregulate caspase-mediated apoptosis.

Our results further indicate that syncytial death in NSCLC cells infected with fusogenic rVSV-NDV proceeds via distinct and

temporally regulated pathways. While A549 cells displayed delayed PS exposure and gradual necrotic collapse, H1437 cells demonstrated a more rapid progression to secondary necrosis. Interestingly, both rVSV-NDV and rVSV induced caspase-mediated apoptosis that coincided with early stages of infection, while the final execution phase is likely correlated with a shift toward necrosis via regulated mechanisms. Although the commercial PS exposure assay used in this study was prone to high inter-experimental variability, the trends were consistent with morphological observations and were reproducible across replicates. To confirm these findings, alternative and complimentary assays could be performed, such as flow cytometry using Annexin V/PI staining or live cell imaging with apoptosis and necrosis reporters, to further interrogate the interplay between apoptotic signaling and necrotic execution in virus-mediated syncytial collapse.

Although not the focus of the current study, we speculate that a differential antiviral response program in H1437 and A549 cells could contribute to the different susceptibilities of these cell lines to rVSV and rVSV-NDV. While H1437 cells have not been previously tested in the context of virus-mediated oncolysis, this hypothesis would explain the rapid replication and killing by VSV in A549 cells before they mount a protective antiviral response,[23] while the slower replicating rVSV-NDV presumably allows enough time for antiviral signaling to occur before complete destruction of the cell monolayer by the virus. In the latter scenario, rVSV-NDV-infected syncytia shed off the monolayer as dead cells, leaving behind surrounding uninfected cells that may have attained an antiviral state. Interestingly, a similar observation of syncytial detachment in A549 cells had been reported previously in the context of respiratory syncytial virus (RSV) infection.[35]

OV-mediated syncytia are phenotypically abnormal,[6] and the expanding networks of rVSV-NDV-infected fused cells are in stark contrast with the shrinking, rounding morphology of rVSV-infected cells. Nevertheless, our results suggest that some of the ability to activate apoptotic signaling had been conferred from rVSV to the chimeric rVSV-NDV, and additional features of a syncytial death may be inherent in the exchanged surface proteins with those of NDV. This hypothesis is supported by the similarities we observed in the dynamic engagement of intrinsic and extrinsic apoptosis between a non-fusogenic and a fusogenic oncolysis. Irrespective of particular, cell line-specific differences, for example in MOMP, caspase activity, or PARP cleavage, our data are in line with reports of likewise dynamic apoptosis activation kinetic caused by rVSV[12–14,31] or oncolytic NDV: Different NDV strains have been reported to activate extrinsic and intrinsic apoptosis, however, with a kinetic during which caspase-8 activation occurred either before,[36,37] in parallel with,[18] or after[38] MOMP and caspase-9 activity. To clarify the ambiguity in the activated apoptotic pathway, we prospectively suggest to investigate the activity of additional initiator or effector caspases, study the interplay of rVSV-NDV with pro- and anti-apoptotic members of the BH-3-only protein family and monitor DNA fragmentation or membrane blebbing as additional hallmarks of apoptosis.

Similar to our rVSV results, we could not identify a molecular mediator determining the switch from apoptosis to necroptosis after rVSV-NDV infection, but we did observe moderate levels of RIPK1, RIPK3, and MLKL expression and phosphorylation in rVSV-NDV-infected NSCLC cells, indicative of syncytial necroptosis as an oncolytic alternative to apoptosis. Our western blot-based necroptosis characterization, however, also indicates a cell line-specific responsiveness, identifying A549 cells as rather necroptosis-reluctant and H1437 cells as moderately responsive to necroptosis stimuli (such as TSZ or 5-Aza-2′-deoxycytidine), primarily due to loss of RIPK3 expression.[24,39–41] However, we successfully triggered necroptotic death in H1437 cells by using a high concentration (100 ng/mL) of TNF-α in the TSZ cocktail[24] (data not shown) and achieved baseline expression levels of necrosome complex proteins determined by western blot in OV-infected A549 and H1437 cells. rVSV-NDV infections in caspase-8 knockout cells have shown that syncytial oncolysis remained unimpaired (A549) or could even be potentiated (H1437), which argues for a potential switch to necroptosis. However, neither knockout of the downstream necroptosis mediator, MLKL, nor pharmacological inhibition of RIP1, RIPK3, or MLKL (data not shown) had a quantifiable effect on the progression of syncytial oncolysis. We thus conclude that rVSV-NDV-mediated syncytial death may not depend on necroptosis and may be complexly regulated. Due to overlapping regulatory roles of RIPK1 and RIPK3 in, for example, apoptotic signaling (extensively reviewed in Wegner et al.[42]), the observed muted death in H1437 RIPK1$^{-/-}$ or H1437 and A549 RIPK3$^{-/-}$ cells could not conclusively define necroptosis dependency during a fusogenic death. This complex necroptosis regulation has also been reported by Liao et al.,[19] who identified a dual regulatory role of RIPK1 during necroptosis of fused, NDV-infected cells. On the one hand, RIPK1 was found to induce RIPK3-MLKL interaction and necroptotic death, but, on the other hand, the authors identified RIPK1 in counteracting necroptosis by recruiting and degrading *p*-MLKL in stress granules.[19]

Independent of the cell line and species, we observed extended syncytiogenesis and prolonged viability in cells that are devoid of caspase-3 activity, which led us to the hypothesis that caspase-3 plays a central role during rVSV-NDV-induced syncytia disintegration. In support of this theory, previous research with syncytia-forming oncolytic RSV or fusogenic Sendai virus similarly described a caspase-3-dependent apoptotic death, which could be prevented by addition of zVAD.[35,43] Although delayed, rVSV-NDV-induced syncytia eventually led to collapse in caspase-3$^{-/-}$ cells. In line with knowledge from syncytia-forming reovirus fusion-associated small transmembrane (FAST) proteins,[44] we thus propose that, during syncytia formation and death, rVSV-NDV maintained membrane integrity for prolonged syncytium expansion, until effector caspases trigger syncytium disassembly.

Our initial pathway characterization focused on two well-characterized cell death modalities, apoptosis and necroptosis. It should be noted, however, that other regulated cell death modes, such as pyroptosis or autophagy, may also play important roles in oncolysis

and syncytia membrane dynamics. Both parental viruses (rVSV and rNDV) have been shown to induce the formation of the NLRP3 inflammasome, leading to gasdermin (GSDM)-controlled pyroptotic death and a pro-inflammatory anti-tumor immune response.[15,45] Furthermore, syncytial death of severe acute respiratory syndrome coronavirus 2-infected epithelial cells has been reported to depend on the activity of caspase-9 and caspase-3 to cleave pyroptosis effector GSDM, since GSDM knockout cells as well as zVAD treatment both abrogated syncytia destruction.[46] It is thus plausible that pyroptosis effector functions, in particular members of the GSDM family, also lead to pore formation after rVSV-NDV-mediated syncytia formation and may be causative for ICD marker release and pro-inflammatory oncolysis. In fact, having seen parallel activation of apoptosis and necroptosis by rVSV-NDV and no clear evidence on the origin of the immunogenicity of cell death, we hypothesize a combined death modality including pyroptosis, which has recently been described as PANoptosis (reviewed in detail in Zhu et al.[47]). While these investigations were beyond the scope of the current work, additional research in this direction is warranted.

Beside the direct oncolytic effect, OVs ignite an inflammatory immune response in the tumor microenvironment, primarily through the release of tumor-associated antigens and immune-stimulating DAMPs and pathogen-associated molecular patterns, which ultimately launches a systemic cellular response targeted against the tumor in addition to the virus.[1] In agreement with our data from HCC,[2] the current study confirms a potent expression and release of canonical ICD markers (HMGB1, ATP, HSP70, HSP90, and CRT) from fusogenic rVSV-NDV-infected human NSCLC cells, and to similar degrees from non-fusogenic rVSV. Considering the heterogeneity in the underlying cell death modality, we could not attribute high levels of immunogenicity to one particular death pathway. However, we report that only the UV-inactivated oncolysates derived from rVSV-NDV-infected A549 or H1437 cells were efficient in activating human DCs (CD86 ^{high}, MHC-I ^{high}, and MHC-II ^{high}) *in vitro*, which agrees with our previous findings of an immunogenic oncolysis that triggered DC activation *in vitro* and in a murine *in vivo* tumor model.[4,25]

In a previous study by our group, we demonstrated synergism and potent, immune-mediated tumor cell killing by combining rVSV-NDV treatment with adoptive T cell transfer in an immuno-competent B16-OVA melanoma mouse model.[3] rVSV-NDV not only modulated tumor immune suppression by upregulation of MHC-I and suppressing PD-L1 on tumors but also successfully recruited $CD8^+$ OVA-antigen-specific OT1 T cells to the tumor sites.[3] We additionally showed that rVSV-NDV therapy synergized with systemic immune checkpoint inhibition with anti-programmed cell death ligand 1,[4] further supporting the potent activation of immune responses by oncolytic virotherapy with this vector. Investigation into ICDs within the context of human NSCLC allowed us to broaden our hypothesis that rVSV-NDV as an immunogenic virus that is capable of heating up the tumor microenvironment and potentially enabling synergism with other immunotherapies, like immune checkpoint inhibitors and CAR T cells.

This encouraging observation confirms previously reported immune-stimulatory properties of a syncytial death.[4,5,10,48] That increased syncytia formation correlates with greater immunogenicity has also been recently shown by Nelson and colleagues,[10] who pseudotyped non-fusogenic rVSV with FAST proteins and tested synergistic effects with NKT cell activation therapy in an aggressive breast cancer model. Interestingly, their study showed superior ICD and better therapeutic responsiveness at even reduced viral doses, compared with less fusogenic FAST-expressing rVSV vectors.[10] However, the question of what determines immunogenicity during syncytial destruction compared with a non-fusogenic cell death remains unresolved. We currently speculate that slower syncytial death kinetics may yield a greater net quantity of immunogenic material, which may be better perceived by surrounding immune cells than antigenic and immune-stimulatory factors from rapidly shrinking, rVSV-infected cells.

In summary, this study unraveled some of the mechanistic complexity underlying syncytial death mediated by the oncolytic hybrid virus rVSV-NDV. We identified a dynamic engagement of an immunogenic type of apoptosis along with necroptosis during fusogenic oncolysis and propose caspase-3 as important mediator in syncytial destruction. However, in the analyzed cell lines, we found most mechanistic signatures overlapping with the death program of parental rVSV. In the need to develop powerful next-generation cancer immunotherapies, an expanded death pathway characterization is needed, as well as the identification of druggable death regulators that could synergize with the oncolytic and immunogenic effects of rVSV-NDV for future clinical translation.

MATERIALS AND METHODS

Cell culture and viruses

All cell culture maintenance and experiments were performed in medium without antibiotics. A549 and AGE1.CR.pIX (ProBioGen) cells were cultured in DMEM/F-12 (Thermo Fisher Scientific) containing 5% fetal calf serum (FCS). H1437 cells were cultured in RPMI-1640 medium containing 10% FCS. B16-OVA cells, and HEK-293T cells were cultured in DMEM-GlutaMAX (Thermo Fisher Scientific) with 10% FCS. BHK-21 cells were cultured in GMEM (Thermo Fisher Scientific) containing 10% FCS and 2% tryptose-phosphate broth. A549 cells have been previously described to have a somewhat functional antiviral (type I IFN) signaling pathway.[49,50] Less is known about antiviral signaling in H1437, but our own data indicate that these pathways are more impaired than A549.

rVSV-NDV and rVSV-NDV-GFP were generated in adherent AGE1.CR.pIX cells and purified by ultracentrifugation over a sucrose gradient as previously described.[2,3] rVSV and rVSV-GFP stocks were generated in adherent BHK-21 cells and purified as previously described.[51]

Viral growth curves and oncolysis measurement

Human or murine cancer cell lines were plated in 6-well, 12-well, or 96-well plates and infected the next day (80%–90% confluence) at various MOIs as specified in the results section. Infections were monitored with an Axiovert 40 CFL Fluorescent microscope (Zeiss), and infectious viral titers were determined from the supernatant at defined timepoints by 50% tissue culture infectious dose ($TCID_{50}$) assay in AGE1.CR.pIX cells and expressed as $TCID_{50}$/mL. Virus-mediated oncolysis was either measured from fresh supernatants using a Cytotox96 non-radioactive cytotoxicity assay (LDH release assay) or measured from infected cells using a CellTiter-Glo luminescent cell viability assay, following the manufacturer's protocols (Promega). Data were recorded on a GloMax Discover microplate reader (Promega).

Real-time quantification of OV-induced cell-cell fusion

To generate reporter cell lines expressing either of the split NanoGlo luciferase, A549 and H1437 cells were transfected with CMV HaloTag-LgBiT or CMV HaloTag-HiBiT vectors (obtained on a limited user license agreement from Promega), respectively, using FuGENE HD transfection reagent (Promega). After 3 weeks of hygromycin-B selection, the respective, stably transfected LgBiT- or HiBiT-expressing cells were co-cultured in a 1:1 ratio in white 96-well plates at 1×10^4 cells/well and infected the next day with rVSV or rVSV-NDV. NanoGlo Endurazine Live Cell Substrate was supplemented in the media, and luminescence, as a measure of cell-cell fusion, was recorded in real time on a GloMax Discover system. To reduce unspecific background from extracellular HiBiT or LgBiT that had leaked from lysed, but non-fused, cells, DrkBiT peptide (Promega) was supplemented as a cell-impermeable, competitive LgBiT inhibitor.

Pharmacological inhibition of cell death

Treatment with human TNF-α (500 ng/mL, Peprotech, Thermo Fisher Scientific) and the Smac mimetic compound (LCL-161; 1 μM, Cayman Chemicals), named TS, was used to induce apoptosis. Staurosporine (10 μM, Cayman Chemicals) served as a broad-spectrum positive control for an apoptotic death. TS and the pan-caspase inhibitor zVAD-fmk (shortened zVAD; 50 μM, AbMole BioScience), named TSZ, was used to induce necroptosis. Mitoxantrone (MTX, 2 μM, Bio-Techne) or doxorubicin (2 μM, Cayman Chemicals) were applied as positive controls for the induction of ICD.

Real-time monitoring of apoptotic cell death

A549 or H1437 cells were seeded in opaque white 96-well plates at $5–10 \times 10^3$ cells/well and infected the next day at an MOI of 0.01. PS exposure and secondary necrosis were measured in real-time using the RealTime-Glo Annexin V Apoptosis and Necrosis assay following the manufacturer's protocols (Promega).

MOMP assay

A549 or H1437 cells were seeded at a density of 1×10^4 cells/well in opaque black 96-well plates. The next day, cells were infected at a series of MOIs, and at 36 hpi, mitochondria were stained with 500 nM of the mitochondria-intercalating dye, TMRE (AAT Bioquest), for 30 min in the dark. Loss of fluorescence signal intensity was measured on a microplate reader at 530/580 nm (excitation/emission) wavelength and attributed to MOMP. Cells treated with the decoupling agent FCCP (2–50 μM, Cayman Chemicals) for 10 min were used as positive controls.

Caspase activity assays

A549 or H1437 cells were seeded in opaque white 96-well plates at $5–10 \times 10^3$ cells/well and infected the next day at an MOI of 1. Caspase activity in infected cells was measured at defined timepoints using Caspase-Glo 3/7 Assay or Caspase-Glo 8 Assay (Promega) following the manufacturer's instructions.

CRISPR-Cas9-mediated gene editing

Target sequences for gene knockouts were designed using CHOPCHOP,[52] and oligonucleotides were obtained from Eurofins Genomics (listed in Table S1). For each human target gene (*caspase-3*, *caspase-8*, *MLKL*, *RIPK1*, and *RIPK3*), a pool of three individual guide RNAs was designed to target at least two exons. Two NTCs (NTC1 and NTC2) single guide RNAs (sgRNAs) were generated. The plentiCRISPR v2 vector (# 52961, AddGene), was kindly provided by Prof. Andreas Pichlmair (Institute of Virology, TUM). Target-specific lentiviral vectors were obtained by sticky-end ligation of pooled, annealed sgRNAs into the linearized plentiCRISPR v2 vector, as previously described,[53,54] followed by transformation of One Shot Stbl3 competent *E. coli* (Invitrogen). Transformed clones were validated by Sanger sequencing using a standard U6 forward primer. For generation of lentivirus, HEK293-T cells were transfected with the sgRNA-containing plentiCRISPR v2 plasmid and psPAX2 (# 12260, Addgene), and pMD2.G (# 12259, Addgene) packaging plasmids using 1 μg/μL polyethyleneimine (Thermo Fisher Scientific) in serum-free media. On days 2–4 after transfection, lentivirus was harvested from the supernatant. Confluent monolayers of A549 or H1437 target cells were transduced with 500 μL of lentiviral supernatant and 10 μg/mL polybrene using spin-infection at 1,000×*g* at 37°C for 90 min. Selection was started on day 2 after transduction using 5 μg/mL puromycin. Puromycin-resistant stably transduced cells were subjected to single-cell clone isolation by limiting dilution. Cells transduced with lentivirus encoding for NTC1 or NTC2 were prepared in a similar manner but used as a polyclonal cell population. Gene knockouts were validated by western blot or exon PCR and DNA sequencing, as previously described.[55] In brief, DNA was isolated from clonal cell pellets and purified by standard isopropanol precipitation. Using primers flanking the targeted exons (Table S2), DNA was PCR amplified and analyzed by agarose gel electrophoresis. Successful gene manipulation was further confirmed by Sanger sequencing over the CRISPR-targeted exons. B16-OVA cells with gene knockouts for caspase-3 and MLKL were kindly provided and validated by Prof. Simon Heidegger (Department of Medicine III, Klinikum rechts der Isar, TUM).

Immunofluorescent staining

A549 or H1437 cells were seeded in 8-well chamber slides (BD Falcon, Franklin Lakes, NJ) at a density of 5×10^4 cells/well. The following day, duplicate wells were infected with rVSV-GFP or

rVSV-NDV-GFP at an MOI of 0.01 or left uninfected. After 24 or 48 h, cells were fixed with 4% paraformaldehyde (PFA) and then subjected to immunofluorescent staining using a rabbit anti-cleaved caspase-3 antibody (Cell Signaling Technology) and Alexa Fluor 647-conjugated AffiniPure goat anti-rabbit IgG (Jackson ImmunoResearch). Slides were mounted in mounting medium containing DAPI (Abcam) for visualization of nuclei. Analysis was performed on an Axio Imager fluorescence microscope (Zeiss), and representative images were captured at 200× magnification.

Immunoblotting

Whole-cell protein lysates were generated using radioimmunoprecipitation assay (RIPA) buffer supplemented with protease inhibitors (cOmplete Protease inhibitor cocktail, Roche) and phosphatase inhibitors (PhosSTOP phosphatase inhibitor cocktail, Roche), and total protein content in cleared lysates was determined by DC Protein Assay (BioRad) against a BSA standard. We separated 20 μg of heat-denatured total protein by SDS-PAGE on 7%, 10%, 12%, or 16% tricine gels, followed by wet-transfer onto 0.2-μm nitrocellulose membranes (BioRad). ROTI-Mark TRICOLOR Protein marker (Carl Roth) was used as a molecular weight marker. Membranes were blocked using 5% skim milk powder (Carl Roth) in Tris-buffered saline containing 0.1% Tween 20 (TBST) for at least 1 h at room temperature and incubated with the following primary antibodies in 2.5% BSA in TBST at 4°C overnight: mouse anti- caspase-3 (31A1067) (#56053, Santa Cruz Biotechnology), mouse anti- caspase-8 (1C12) (#9746, Cell Signaling Technology [CST]), rabbit anti-caspase-9 (#9502, CST), rabbit anti-cleaved caspase-9 (D315) (#9505, CST), rabbit anti-PARP antibody (#9542, CST), rabbit anti-human phospho RipK1 (S166) (D1L3S) (#65746, CST), mouse anti-RIP (clone 38/RIP) (#610459, BD Biosciences), rabbit anti-human RIPK3 antibody (#226297, Abcam), rabbit anti-RIPK3 (phospho S227) (EPR 9627) (#209384, Abcam), rabbit anti-MLKL (phospho S358) antibody [EPR9514] (#187091, Abcam), and rabbit anti-MLKL antibody [EPR17514] (#184718, Abcam). After at least six washing steps (10 min each) using TBST, blots were incubated in HRP-linked secondary antibodies (horse anti-mouse immunoglobulin G [IgG], goat anti-rabbit IgG, #7076, #7074, CST) for at least 1 h at room temperature, and membranes were recorded with a Gel Doc XR + Documentation system and ImageLab software (BioRad). Membranes were stripped using Restore Plus stripping buffer (Thermo Fisher Scientific), re-blocked with milk for at least 1 h, and incubated with loading control antibodies rabbit anti-GAPDH (14C10), (#2118, CST) or mouse anti-β-actin clone AC-74 (#A2228, Merck), or another primary antibody. Protein was quantified using densitometry within ImageLab software, accounting for background signal subtraction and expressing the protein intensity relative to the signal of the loading control. The obtained normalized volume intensities were compared relative to protein quantities in untreated samples and averaged over the biological replicates.

Quantification of released ICD markers

Cells were seeded in six-well plates and, the next day, infected at an MOI of 0.01 using phenol red-free and serum-free DMEM supplemented with 2.5 mM glutamine. At 24, 48, and 72 hpi, the culture media were collected and precleared from cell debris by centrifugation at 1,000×*g* for 5 min at 4°C, followed by a 30-fold concentration at 4000×*g* for 30–45 min at 4°C using Amicon Ultra-2 centrifugal filters with a 10-kD cutoff (Merck). Concentrated supernatant was supplemented with protease and phosphatase inhibitors, and 20 μg total protein (determined by *DC* Protein assay; BioRad) was analyzed by SDS-PAGE and western blotting. Whole-cell RIPA lysates collected at 24 hpi served as controls. Membranes were incubated with primary antibodies detecting mouse anti-HSP70 (3A3) (#32239, Santa Cruz), rabbit anti-HSP90 (#4874, CST), rabbit anti-CRT (D3E6) XP (#12238, CST), rabbit anti-HMGB1 (D3E5) (#6893, CST), and processed as described above. Protein was quantified relative to a total protein Ponceau-S staining using densitometry within ImageLab software.

To quantify released ATP, cells were seeded in 12-well plates and infected the next day at an MOI of 0.01 in serum-free media. Culture supernatants were collected at defined time points, and released ATP was directly quantified from the fresh supernatants with the ATP Bioluminescence Assay Kit HS II (Roche).

Flow cytometry quantification of CRT cell surface exposure

Cells were seeded in 12-well plates and infected the next day at various MOIs and harvested at 24 hpi using Trypsin/EDTA. At least 1×10^5 cells were transferred to a 96-well V-bottom fluorescence-activated cell sorting (FACS) plate for cell surface staining of CRT, similar to a previously described method.[56] Briefly, cells were washed with PBS and first stained with Viobility 405/520 (Miltenyi Biotec) on ice for 30 min, washed once with PBS, and thereafter stained with rabbit anti-human CRT (ER-marker) antibody (#2907, Abcam) for at least 60 min. Primary staining was followed by a single wash with PBS and fixation of cells in 4% PFA in dH_2O for 15 min on ice. Cells were washed three times and incubated with anti-rabbit Alexa Fluor 488 (#150077, Abcam) secondary antibody for 60 min on ice. After an additional two washing steps with PBS, cell-surface exposure of CRT was measured on a Cytoflex S flow cytometer (Beckman Coulter), and data were analyzed using FlowJo software (BD Biosciences).

Generation of DCs from human PBMCs

PBMCs were isolated from whole blood of healthy male and female human donors (obtained with informed consent, and according to ethics approval #318/19 S-SR by the Ethics Commission of the Technical University of Munich, Germany), and monocytes were *in vitro* differentiated into DCs with human interleukin (IL)-4 (Peprotech) and human granulocyte macrophage colony stimulating factor (GM-CSF) (Biolegend) for 6 days. In brief, white blood cells were isolated from 50 mL of heparinized whole blood diluted 1:1 with PBS using Ficoll gradient (Lymphoprep, Stemcell Technologies) and centrifugation at 1,000×*g* for 30 min at room temperature (no brake). Platelets were removed by two washing steps, and cell debris were removed by passing through a 100 μm cell strainer. 1.5×10^7 PBMCs/well were seeded into six-well plates in very-low endotoxin

(VLE) RPMI media (Gibco) containing 1.5% human serum, and 3–4 h later, T and B cells were removed with the supernatant. Fresh VLE-RPMI media containing 580 U/mL human IL-4 (Peprotech) and 800 U/mL human GM-CSF (Biolegend) was added, and PBMCs were differentiated for 6 days to obtain cDCs consistent with the cDC2 subtype.

In vitro pulsing of human DCs with A549 and H1437 oncolysates

Oncolysates from OV-infected A549 or H1437 cells were harvested at the time of maximum syncytia formation. In detail, cells were infected at an MOI of 0.01 in 12-well plates, and at 48 hpi (H1437) or 72 hpi (A549), cells were washed, scraped off the plate, and live virus in the oncolysate was inactivated for 5 min with UV-B light, before freezing at -80°C. Human cDC2 cells were harvested, and 1×10^5 cells were co-cultured with 100 μL of the UV-inactivated oncolysate in low-binding 96-well flat-bottom plates for 24 h. Non-co-cultured cDC2 cells treated for 48 h with an activation cocktail (2,000 U/mL IL-1-β [Peprotech], 5,000 U/mL IFN-γ [Peprotech], 1 μg/mL R848 [Invivogen], 250 ng/mL prostaglandin E2 [Cayman Chemicals], 20 ng/mL TNF-α, 580 U/mL IL-4, and 800 U/mL GM-CSF) were used as a positive control.

Quantification of DC differentiation and activation by flow cytometry

For flow cytometry analysis of the differentiation and activation status, cocktail-treated or co-cultured DCs were transferred to 96-well V-bottom FACS plates and stained with the following mouse-anti-human antibodies diluted 1:200 in PBS for 30–45 min at 4°C in the dark: CD1a-Pacific Blue (#300124, Biolegend), CD14-PE-Cy7 (clone M5E2) (#557742, BD Bioscience), CD86-PE (#374206, Biolegend), CD141 (BDCA-3)-APC-Vio770 (#130-113-315, Miltenyi Biotec), HLA-DR (MHC-I)-PerCP-Cy5.5 (#311420, Biolegend), HLA-ABC (MHC-II)-BV-605 (#311432, Biolegend). Viobility 405/520 Fixable dye (Miltenyi Biotec) diluted 1:300 in PBS was used for live-dead discrimination. Data were recorded on a Cytoflex S flow cytometer (Beckman Coulter), and compensation was performed based on single-antibody staining with UltraComp eBeads (Thermo Fisher Scientific). FlowJo software (BD Biosciences) was used for analysis. DC subtype determination was performed after IL-4 and GM-CSF differentiation using an alternative panel including the following anti-human antibodies: CD1c (L161), Pacific Blue (#331507, Biolegend); CD11c, APC clone 3.9 ((#301613, Biolegend); CD14, PE-Cy7 (clone M5E2) (#557742, BD Bioscience); CD123, FITC (#306013, Biolegend); HLA-DR (MHC-I) , PerCP-Cy5.5 (#311420, Biolegend); and HLA-ABC (MHC-II), BV-605 (#311432, Biolegend).

Statistical analysis

All data were plotted and analyzed in PRISM version 8.0 (GraphPad, San Diego, CA, USA). Experiments were performed in biological triplicates ($n = 3$, unless otherwise indicated), and mean values with SDs were used for further analysis. An unpaired two-tailed Student's t test was applied for individual data point comparison of two groups, while one-way ANOVA with Tukey's multiple comparisons test was performed for the analysis of more than two groups. p values of less than 0.05 were considered to be statistically significant and are indicated in the graphs.

DATA AVAILABILITY

All raw and analyzed data used in the preparation of this manuscript are available upon request.

ACKNOWLEDGMENTS

The authors would like to thank all volunteering blood donors and Cécilia Lozano-Simon for assistance with PBMC isolation. We want to thank Wei Xie from Peter Vandenabeele's lab at VIB-UGent for providing a protocol for calreticulin flow cytometry. The authors further thank the Department of Virology at TUM for providing the CytoFLEX for flow cytometry analysis and ProBioGen AG (Berlin) for providing the AGE1.CR.pIX cells used for virus production and titration. They also thank Irina Ingold (Department of Internal Medicine III, TUM) for providing the H1437 cells. This work was funded by the European Research Council (ERC) under the European Union's Horizon 2020 Research and Innovation Program (grant agreement #853433), the German Cancer Aid (grant #70113272), and the German Research Foundation (DFG) (grant #519840568).

AUTHOR CONTRIBUTIONS

Conceptualization, J.A.; methodology, J.A., F.K., N.H., A.B., and S.G.; investigation, F.K. N.H., formal analysis, F.K., N.H., A.B., and S.G., data curation and writing original draft, F.K.; writing – review & editing, J.A.; supervision, J.A.; project administration, J.A.; funding acquisition, J.A. All authors have read and agreed to the published version of the manuscript.

DECLARATION OF INTERESTS

J.A. is a patent holder for the development and use of rVSV-NDV as an oncolytic therapy of cancer and is co-founder of Fusix Biotech GmbH, which is developing the rVSV-NDV technology for clinical use.

SUPPLEMENTAL INFORMATION

Supplemental information can be found online at https://doi.org/10.1016/j.omton.2025.201027.

REFERENCES

1. Palanivelu, L., Liu, C.H., and Lin, L.T. (2022). Immunogenic cell death: The cornerstone of oncolytic viro-immunotherapy. Front. Immunol. *13*, 1038226. https://doi.org/10.3389/fimmu.2022.1038226.
2. Abdullahi, S., Jakel, M., Behrend, S.J., Steiger, K., Topping, G., Krabbe, T., Colombo, A., Sandig, V., Schiergens, T.S., Thasler, W.E., et al. (2018). A Novel Chimeric Oncolytic Virus Vector for Improved Safety and Efficacy as a Platform for the Treatment of Hepatocellular Carcinoma. J. Virol. *92*, e01386-18. https://doi.org/10.1128/JVI.01386-18.
3. Krabbe, T., Marek, J., Groll, T., Steiger, K., Schmid, R.M., Krackhardt, A.M., and Altomonte, J. (2021). Adoptive T Cell Therapy Is Complemented by Oncolytic Virotherapy with Fusogenic VSV-NDV in Combination Treatment of Murine Melanoma. Cancers (Basel) *13*, 1044. https://doi.org/10.3390/cancers13051044.
4. Marek, J., Hanesch, L., Krabbe, T., El Khawanky, N., Heidegger, S., and Altomonte, J. (2023). Oncolytic virotherapy with chimeric VSV-NDV synergistically supports RIG-I-dependent checkpoint inhibitor immunotherapy. Mol. Ther. Oncolytics *30*, 117–131. https://doi.org/10.1016/j.omto.2023.08.001.
5. Bateman, A.R., Harrington, K.J., Kottke, T., Ahmed, A., Melcher, A.A., Gough, M.J., Linardakis, E., Riddle, D., Dietz, A., Lohse, C.M., et al. (2002). Viral fusogenic membrane glycoproteins kill solid tumor cells by nonapoptotic mechanisms that promote cross presentation of tumor antigens by dendritic cells. Cancer Res. *62*, 6566–6578.
6. Burton, C., and Bartee, E. (2019). Syncytia Formation in Oncolytic Virotherapy. Mol. Ther. Oncolytics *15*, 131–139. https://doi.org/10.1016/j.omto.2019.09.006.
7. Krabbe, T., and Altomonte, J. (2018). Fusogenic Viruses in Oncolytic Immunotherapy. Cancers (Basel) *10*, 216. https://doi.org/10.3390/cancers10070216.

8. Nakatake, M., Kuwano, N., Kaitsurumaru, E., Kurosaki, H., and Nakamura, T. (2021). Fusogenic oncolytic vaccinia virus enhances systemic antitumor immune response by modulating the tumor microenvironment. Mol. Ther. *29*, 1782–1793. https://doi.org/10.1016/j.ymthe.2020.12.024.

9. Zaher, K.A., Alrahimi, J.S., Basingab, F.S., and Aldahlawi, A.M. (2024). Newcastle Disease Virus Virotherapy: Unveiling Oncolytic Efficacy and Immunomodulation. Biomedicines *12*, 1497. https://doi.org/10.3390/biomedicines12071497.

10. Nelson, A., McMullen, N., Gebremeskel, S., De Antueno, R., Mackenzie, D., Duncan, R., and Johnston, B. (2024). Fusogenic vesicular stomatitis virus combined with natural killer T cell immunotherapy controls metastatic breast cancer. Breast Cancer Res. *26*, 78. https://doi.org/10.1186/s13058-024-01818-5.

11. Le Boeuf, F., Gebremeskel, S., McMullen, N., He, H., Greenshields, A.L., Hoskin, D. W., Bell, J.C., Johnston, B., Pan, C., and Duncan, R. (2017). Reovirus FAST Protein Enhances Vesicular Stomatitis Virus Oncolytic Virotherapy in Primary and Metastatic Tumor Models. Mol. Ther. Oncolytics *6*, 80–89. https://doi.org/10.1016/j.omto.2017.08.001.

12. Schache, P., Gürlevik, E., Strüver, N., Woller, N., Malek, N., Zender, L., Manns, M., Wirth, T., Kühnel, F., and Kubicka, S. (2009). VSV virotherapy improves chemotherapy by triggering apoptosis due to proteasomal degradation of Mcl-1. Gene Ther. *16*, 849–861. https://doi.org/10.1038/gt.2009.39.

13. Felt, S.A., Moerdyk-Schauwecker, M.J., and Grdzelishvili, V.Z. (2015). Induction of apoptosis in pancreatic cancer cells by vesicular stomatitis virus. Virology *474*, 163–173. https://doi.org/10.1016/j.virol.2014.10.026.

14. Cary, Z.D., Willingham, M.C., and Lyles, D.S. (2011). Oncolytic vesicular stomatitis virus induces apoptosis in U87 glioblastoma cells by a type II death receptor mechanism and induces cell death and tumor clearance in vivo. J. Virol. *85*, 5708–5717. https://doi.org/10.1128/jvi.02393-10.

15. Wang, B., Zhu, J., Li, D., Wang, Y., Zhan, Y., Tan, L., Qiu, X., Sun, Y., Song, C., Meng, C., et al. (2016). Newcastle disease virus infection induces activation of the NLRP3 inflammasome. Virology *496*, 90–96. https://doi.org/10.1016/j.virol.2016.05.023.

16. Ye, T., Jiang, K., Wei, L., Barr, M.P., Xu, Q., Zhang, G., Ding, C., Meng, S., and Piao, H. (2018). Oncolytic Newcastle disease virus induces autophagy-dependent immunogenic cell death in lung cancer cells. Am. J. Cancer Res. 8, 1514–1527.

17. Mansour, M., Palese, P., and Zamarin, D. (2011). Oncolytic specificity of Newcastle disease virus is mediated by selectivity for apoptosis-resistant cells. J. Virol. *85*, 6015–6023. https://doi.org/10.1128/jvi.01537-10.

18. Cuadrado-Castano, S., Ayllon, J., Mansour, M., de la Iglesia-Vicente, J., Jordan, S., Tripathi, S., García-Sastre, A., and Villar, E. (2015). Enhancement of the proapoptotic properties of Newcastle disease virus promotes tumor remission in syngeneic murine cancer models. Mol. Cancer Ther. *14*, 1247–1258. https://doi.org/10.1158/1535-7163.Mct-14-0913.

19. Liao, Y., Wang, H.X., Mao, X., Fang, H., Wang, H., Li, Y., Sun, Y., Meng, C., Tan, L., Song, C., et al. (2017). RIP1 is a central signaling protein in regulation of TNF-α/TRAIL mediated apoptosis and necroptosis during Newcastle disease virus infection. Oncotarget *8*, 43201–43217. https://doi.org/10.18632/oncotarget.17970.

20. Chen, J., Kos, R., Garssen, J., and Redegeld, F. (2019). Molecular Insights into the Mechanism of Necroptosis: The Necrosome As a Potential Therapeutic Target. Cells *8*, 1486. https://doi.org/10.3390/cells8121486.

21. Stewart, G., Tazzyman, S., Sun, Y., Andrews, R.E., Harrison, J., Lath, D., Down, J., Robinson, G., Wang, X., Muthana, M., et al. (2025). An oncolytic adenovirus targeting SLAMF7 demonstrates anti-myeloma efficacy. Leukemia *39*, 1449–1463. https://doi.org/10.1038/s41375-025-02617-3.

22. Kim, S.E., Bak, S., Park, J.H., Lee, J.E., Lee, Y.J., Park, M.S., Ko, S., Choi, H., Jung, K. H., Na, K., and Hong, S.S. (2025). CD44-targeted RIP3 gene delivery strategy for enhanced cancer therapy via necroptosis and immune modulation. J. Control. Release *384*, 113872. https://doi.org/10.1016/j.jconrel.2025.113872.

23. Voigt, E.A., Swick, A., and Yin, J. (2016). Rapid induction and persistence of paracrine-induced cellular antiviral states arrest viral infection spread in A549 cells. Virology *496*, 59–66. https://doi.org/10.1016/j.virol.2016.05.019.

24. Najafov, A., Zervantonakis, I.K., Mookhtiar, A.K., Greninger, P., March, R.J., Egan, R.K., Luu, H.S., Stover, D.G., Matulonis, U.A., Benes, C.H., and Yuan, J. (2018). BRAF and AXL oncogenes drive RIPK3 expression loss in cancer. PLoS Biol. *16*, e2005756. https://doi.org/10.1371/journal.pbio.2005756.

25. Glauss, S., Neumeyer, V., Hanesch, L., Marek, J., Hartmann, N., Wiedemann, G.M., and Altomonte, J. (2024). A Novel Chimeric Oncolytic Virus Mediates a Multifaceted Cellular Immune Response in a Syngeneic B16 Melanoma Model. Cancers (Basel) *16*, 3405. https://doi.org/10.3390/cancers16193405.

26. Obeid, M., Tesniere, A., Ghiringhelli, F., Fimia, G.M., Apetoh, L., Perfettini, J.L., Castedo, M., Mignot, G., Panaretakis, T., Casares, N., et al. (2007). Calreticulin exposure dictates the immunogenicity of cancer cell death. Nat. Med. *13*, 54–61. https://doi.org/10.1038/nm1523.

27. Chen, D.S., and Mellman, I. (2013). Oncology Meets Immunology: The Cancer-Immunity Cycle. Immunity *39*, 1–10. https://doi.org/10.1016/j.immuni.2013.07.012.

28. Lamberti, M.J., Nigro, A., Mentucci, F.M., Rumie Vittar, N.B., Casolaro, V., and Dal Col, J. (2020). Dendritic Cells and Immunogenic Cancer Cell Death: A Combination for Improving Antitumor Immunity. Pharmaceutics *12*, 256. https://doi.org/10.3390/pharmaceutics12030256.

29. Fucikova, J., Kepp, O., Kasikova, L., Petroni, G., Yamazaki, T., Liu, P., Zhao, L., Spisek, R., Kroemer, G., and Galluzzi, L. (2020). Detection of immunogenic cell death and its relevance for cancer therapy. Cell Death Dis. *11*, 1013. https://doi.org/10.1038/s41419-020-03221-2.

30. Janssens, S., Rennen, S., and Agostinis, P. (2024). Decoding immunogenic cell death from a dendritic cell perspective. Immunol. Rev. *321*, 350–370. https://doi.org/10.1111/imr.13301.

31. Sharif-Askari, E., Nakhaei, P., Oliere, S., Tumilasci, V., Hernandez, E., Wilkinson, P., Lin, R., Bell, J., and Hiscott, J. (2007). Bax-dependent mitochondrial membrane permeabilization enhances IRF3-mediated innate immune response during VSV infection. Virology *365*, 20–33. https://doi.org/10.1016/j.virol.2007.03.011.

32. Hobbs, J.A., Hommel-Berrey, G., and Brahmi, Z. (2003). Requirement of caspase-3 for efficient apoptosis induction and caspase-7 activation but not viral replication or cell rounding in cells infected with vesicular stomatitis virus. Hum. Immunol. *64*, 82–92. https://doi.org/10.1016/s0198-8859(02)00702-4.

33. da Costa, L.S., Outlioua, A., Anginot, A., Akarid, K., and Arnoult, D. (2019). RNA viruses promote activation of the NLRP3 inflammasome through cytopathogenic effect-induced potassium efflux. Cell Death Dis. *10*, 346. https://doi.org/10.1038/s41419-019-1579-0.

34. Van den Berghe, T., Kaiser, W.J., Bertrand, M.J., and Vandenabeele, P. (2015). Molecular crosstalk between apoptosis, necroptosis, and survival signaling. Mol. Cell. Oncol. *2*, e975093. https://doi.org/10.4161/23723556.2014.975093.

35. Eckardt-Michel, J., Lorek, M., Baxmann, D., Grunwald, T., Keil, G.M., and Zimmer, G. (2008). The fusion protein of respiratory syncytial virus triggers p53-dependent apoptosis. J. Virol. *82*, 3236–3249. https://doi.org/10.1128/jvi.01887-07.

36. Meng, S., Zhou, Z., Chen, F., Kong, X., Liu, H., Jiang, K., Liu, W., Hu, M., Zhang, X., Ding, C., and Wu, Y. (2012). Newcastle disease virus induces apoptosis in cisplatin-resistant human lung adenocarcinoma A549 cells in vitro and in vivo. Cancer Lett. *317*, 56–64. https://doi.org/10.1016/j.canlet.2011.11.008.

37. Ravindra, P.V., Tiwari, A.K., Ratta, B., Bais, M.V., Chaturvedi, U., Palia, S.K., Sharma, B., and Chauhan, R.S. (2009). Time course of Newcastle disease virus-induced apoptotic pathways. Virus Res. *144*, 350–354. https://doi.org/10.1016/j.virusres.2009.05.012.

38. Elankumaran, S., Rockemann, D., and Samal, S.K. (2006). Newcastle disease virus exerts oncolysis by both intrinsic and extrinsic caspase-dependent pathways of cell death. J. Virol. *80*, 7522–7534. https://doi.org/10.1128/jvi.00241-06.

39. Moriwaki, K., Bertin, J., Gough, P.J., Orlowski, G.M., and Chan, F.K.M. (2015). Differential roles of RIPK1 and RIPK3 in TNF-induced necroptosis and chemotherapeutic agent-induced cell death. Cell Death Dis. *6*, e1636. https://doi.org/10.1038/cddis.2015.16.

40. Yang, C., Li, J., Yu, L., Zhang, Z., Xu, F., Jiang, L., Zhou, X., and He, S. (2017). Regulation of RIP3 by the transcription factor Sp1 and the epigenetic regulator UHRF1 modulates cancer cell necroptosis. Cell Death Dis. *8*, e3084. https://doi.org/10.1038/cddis.2017.483.

41. Koo, G.B., Morgan, M.J., Lee, D.G., Kim, W.J., Yoon, J.H., Koo, J.S., Kim, S.I., Kim, S. J., Son, M.K., Hong, S.S., et al. (2015). Methylation-dependent loss of RIP3 expression in cancer represses programmed necrosis in response to chemotherapeutics. Cell Res. *25*, 707–725. https://doi.org/10.1038/cr.2015.56.

42. Wegner, K.W., Saleh, D., and Degterev, A. (2017). Complex Pathologic Roles of RIPK1 and RIPK3: Moving Beyond Necroptosis. Trends Pharmacol. Sci. *38*, 202–225. https://doi.org/10.1016/j.tips.2016.12.005.

43. Miyagawa, Y., Araki, K., Yamashita, T., Tanaka, S., Tanaka, Y., Tomifuji, M., Ueda, Y., Yonemitsu, Y., Shimada, H., and Shiotani, A. (2019). Induction of cell fusion/apoptosis in anaplastic thyroid carcinoma in orthotopic mouse model by urokinase-specific oncolytic Sendai virus. Head Neck *41*, 2873–2882. https://doi.org/10.1002/hed.25769.

44. Salsman, J., Top, D., Boutilier, J., and Duncan, R. (2005). Extensive syncytium formation mediated by the reovirus FAST proteins triggers apoptosis-induced membrane instability. J. Virol. *79*, 8090–8100. https://doi.org/10.1128/jvi.79.13.8090-8100.2005.

45. Lin, J., Liu, F., Gao, F., Chen, Y., Wang, R., Wang, X., Li, Y., Li, Q., Sun, S., Li, Z., et al. (2022). Vesicular stomatitis virus sensitizes immunologically cold tumors to checkpoint blockade by inducing pyroptosis. Biochim. Biophys. Acta. Mol. Basis Dis. *1868*, 166538. https://doi.org/10.1016/j.bbadis.2022.166538.

46. Ma, H., Zhu, Z., Lin, H., Wang, S., Zhang, P., Li, Y., Li, L., Wang, J., Zhao, Y., and Han, J. (2021). Pyroptosis of syncytia formed by fusion of SARS-CoV-2 spike and ACE2-expressing cells. Cell Discov. *7*, 73. https://doi.org/10.1038/s41421-021-00310-0.

47. Zhu, P., Ke, Z.R., Chen, J.X., Li, S.J., Ma, T.L., and Fan, X.L. (2023). Advances in mechanism and regulation of PANoptosis: Prospects in disease treatment. Front. Immunol. *14*, 1120034. https://doi.org/10.3389/fimmu.2023.1120034.

48. Gauvrit, A., Brandler, S., Sapede-Peroz, C., Boisgerault, N., Tangy, F., and Gregoire, M. (2008). Measles virus induces oncolysis of mesothelioma cells and allows dendritic cells to cross-prime tumor-specific CD8 response. Cancer Res. *68*, 4882–4892. https://doi.org/10.1158/0008-5472.Can-07-6265.

49. Lim, H.A., Noh, J.Y., Jang, S.S., Kim, M.C., Moon, S.H., Kim, H.Y., Mun, D.Y., and Kim, H.K. (2023). Innate antiviral responses against Shaan virus infection in HEK293, A549 and MARC-145 cells and limited role of viperin against Shaan virus replication. Heliyon *9*, e22597. https://doi.org/10.1016/j.heliyon.2023.e22597.

50. Buggele, W.A., and Horvath, C.M. (2013). MicroRNA profiling of Sendai virus-infected A549 cells identifies miR-203 as an interferon-inducible regulator of IFIT1/ISG56. J. Virol. *87*, 9260–9270. https://doi.org/10.1128/JVI.01064-13.

51. Altomonte, J., Braren, R., Schulz, S., Marozin, S., Rummeny, E.J., Schmid, R.M., and Ebert, O. (2008). Synergistic antitumor effects of transarterial viroembolization for multifocal hepatocellular carcinoma in rats. Hepatology *48*, 1864–1873. https://doi.org/10.1002/hep.22546.

52. Labun, K., Montague, T.G., Gagnon, J.A., Thyme, S.B., and Valen, E. (2016). CHOPCHOP v2: a web tool for the next generation of CRISPR genome engineering. Nucleic Acids Res. *44*, W272–W276. https://doi.org/10.1093/nar/gkw398.

53. Sanjana, N.E., Shalem, O., and Zhang, F. (2014). Improved vectors and genome-wide libraries for CRISPR screening. Nat. Methods *11*, 783–784. https://doi.org/10.1038/nmeth.3047.

54. Shalem, O., Sanjana, N.E., Hartenian, E., Shi, X., Scott, D.A., Mikkelson, T., Heckl, D., Ebert, B.L., Root, D.E., Doench, J.G., and Zhang, F. (2014). Genome-scale CRISPR-Cas9 knockout screening in human cells. Science *343*, 84–87. https://doi.org/10.1126/science.1247005.

55. Liu, X., Zhou, M., Mei, L., Ruan, J., Hu, Q., Peng, J., Su, H., Liao, H., Liu, S., Liu, W., et al. (2016). Key roles of necroptotic factors in promoting tumor growth. Oncotarget *7*, 22219–22233. https://doi.org/10.18632/oncotarget.7924.

56. Liu, P., Zhao, L., Kepp, O., and Kroemer, G. (2020). Quantitation of calreticulin exposure associated with immunogenic cell death. Methods Enzymol. *632*, 1–13. https://doi.org/10.1016/bs.mie.2019.05.011.

OMTON, Volume *33*

Supplemental information

Syncytial death mediated by oncolytic rVSV-NDV dynamically activates immunogenic apoptosis and necroptosis in human lung cancer cells

Fabian Kortum, Nina Hartmann, Alexander Bryan, Sonja Glauß, and Jennifer Altomonte

Supplemental Tables

Table S1. Single guide RNA sequences and targeted exons for CRISPR/Cas9-mediated gene knockout

Gene target (human)	Ensembl #	sgRNA sequences 5' – 3'	target
Caspase 3	ENST00000308394	AGGAATGACATCTCGGTCTG	Exon 5
		ATGTCGATGCAGCAAACCTC	Exon 5
		GTCGCTTTGTGCCATGCTGA	Exon 8
Caspase 8	ENST00000673742	CCCTCAAGTTCCTGAGCCTG	Exon 2
		CGGGGATACTGTCTGATCAT	Exon 7
		CAAACGAGATATATCCCGGA	Exon 8
MLKL	ENST00000308807	TTGAAGCATATTATCACCCT	Exon 2
		GCATATTATCACCCTTGGCC	Exon 2
		TTAGCTTTGGAATCGTCCTC	Exon 9
RIPK1	ENST00000259808	GTGTTCCAGGATCGACCTTC	Exon 11
		TCTACTACATGGCGCCCGAG	Exon 5
		AGCACCTGAATGACGTCAAC	Exon 5
RIPK3	ENST00000216274	CCGCCCCCTTGGTGTCCATC	Exon 2
		CCTTGGTGTCCATCGAGGAA	Exon 2
		TCACTCGTGTACGAAGCAGT	Exon 6
NTC1 (from [44])	/	ACGGAGGCTAAGCGTCGCAA	/
NTC2 (from [44])	/	CGCTTCCGCGGCCCGTTCAA	/

Table S2. Forward and reverse primer sequences

Primer name	Application	Sequence 5' – 3'	Expected fragment [bp]
RIPK3_exon2_fwd	Primer for CRISPR/Cas9 knockout validation (flanking exon 2 of RIPK3)	CCAGCCTGATGTCGTGCGTCAA	450
RIPK3_exon2_rev		ATTCAGGCCCCAGAGCACAGTG	
RIPK3_exon6_fwd	Primer for CRISPR/Cas9 knockout validation (flanking exon 6 of RIPK3)	GGATCCTTAACCCCAGGTGCCCTT	460
RIPK3_exon6_rev		AGGGACAGCAGAGTGGAGTGCA	
MLKL_exon9_fwd	Primer for CRISPR/Cas9 knockout validation (flanking exon 9 of MLKL)	TCAAACTCCTCCAAGGACCACC	488
MLKL_exon9_rev		GCCACCAGCTTGCGGATCTTCT	
MLKL_exon2_fwd	Primer for CRISPR/Cas9 knockout validation (flanking exon 2 of MLKL)	TGGACCAGCTCTTCCCAGCTACA	436
MLKL_exon2_rev		ATCTCCCCATTAGCCTCCTCCAGG	

Abbreviations: fwd (forward); rev (reverse); bp (base pairs)

Supplemental Figures

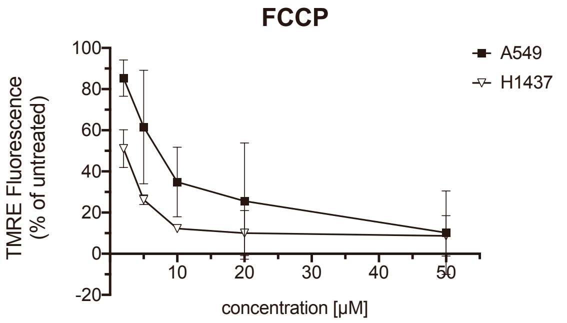

Figure S1: MOMP induced by the uncoupling agent FCCP in A549 and H1437 cells

Permeabilization of the outer mitochondrial membrane (MOMP) was mimicked by the uncoupling agent, FCCP at 2, 5, 10, 20, 50 µM. FCCP was added for 10 min, and mitochondria were then stained with TMRE fluorescent dye. Data are shown as % fluorescence reduction relative to untreated cells.

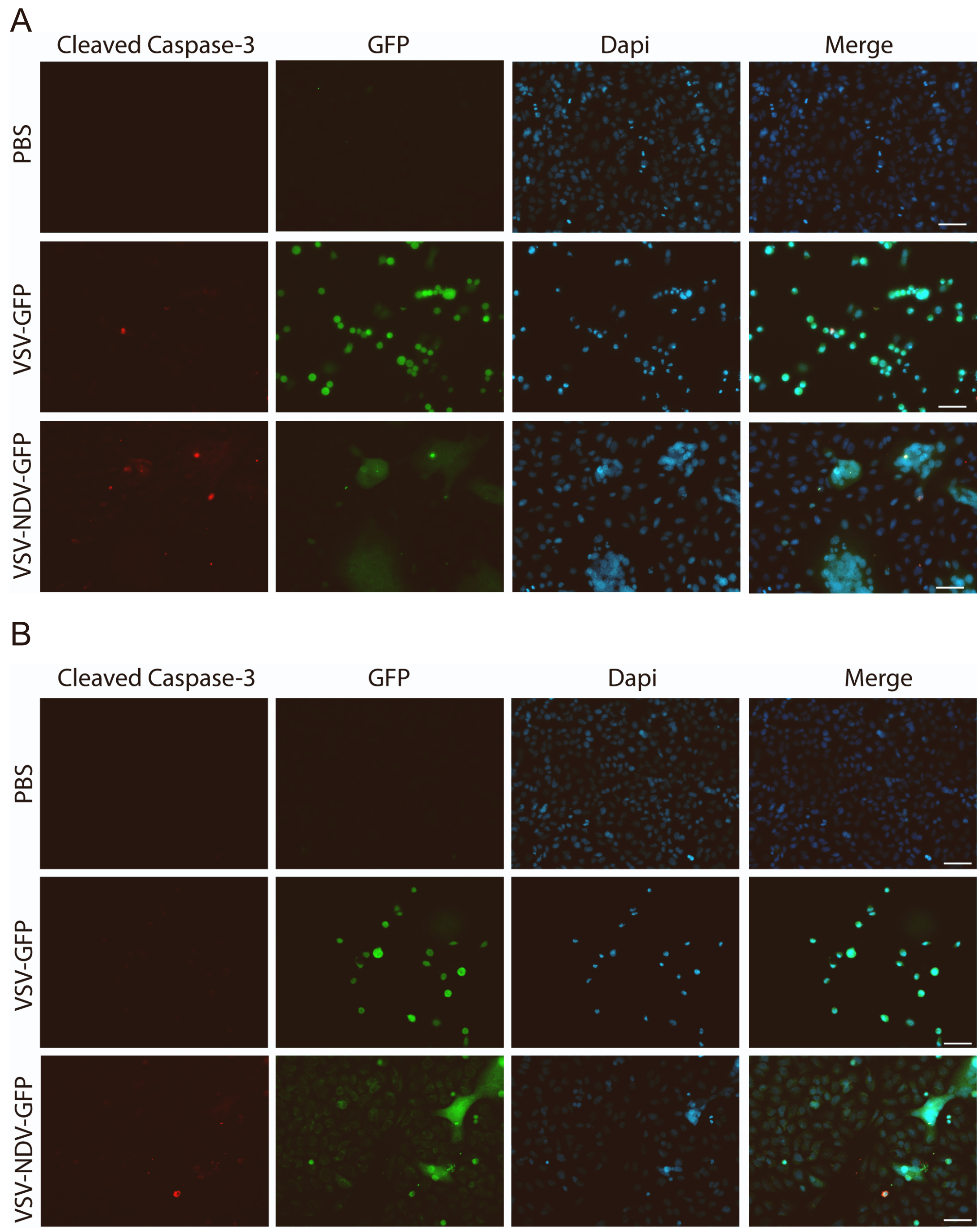

Figure S2: Cleaved Caspase-3 in VSV- or VSV-NDV-infected A549 cells. A549 cells were infected in chamber slides at an MOI of 0.01 with rVSV-GFP or rVSV-NDV-GFP or left uninfected. (A) After 24 or (B) 48 hours, cells were fixed and subjected to immunofluorescent staining for cleaved caspase-3. Nuclei were visualized by DAPI staining. Cells were analyzed by fluorescence microscopy, and representative images were captured at 200x magnification. Scale bars represent 50μm.

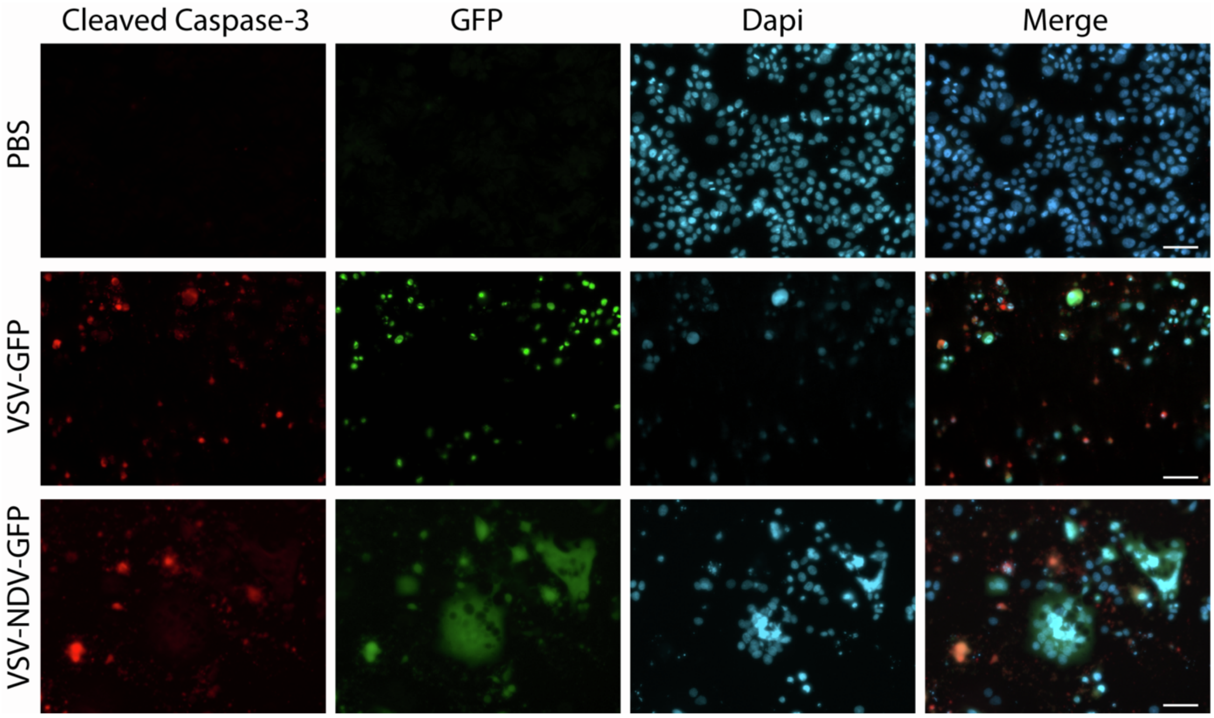

Figure S3: Cleaved Caspase-3 in VSV- or VSV-NDV-infected H1437 cells. H1437 cells were infected in chamber slides at an MOI of 0.01 with rVSV-GFP or rVSV-NDV-GFP or left uninfected. After 24 hours, cells were fixed and subjected to immunofluorescent staining for cleaved caspase-3. Nuclei were visualized by DAPI staining. Cells were analyzed by fluorescence microscopy, and representative images were captured at 200x magnification. Scale bars represent 50μm.

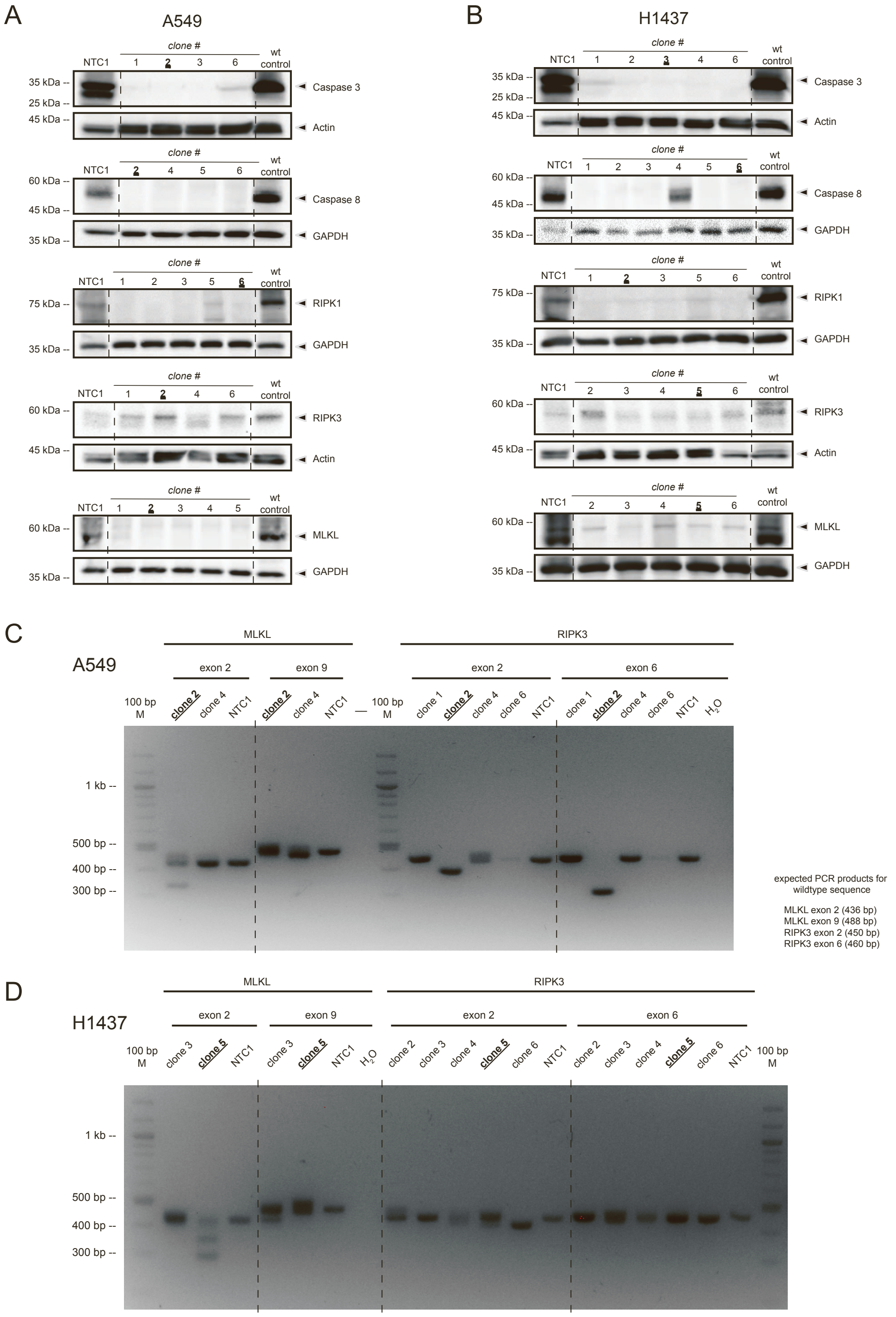

Figure S4: Western blot and exon PCR validation of CRISPR/Cas9 gene knockouts in A549 and H1437 single cell clones

Puromycin-resistant single-cell clones for each target gene were isolated by limiting dilution, and successful gene knockout in (**A**) A549 and (**B**) H1437 cells was validated by Western blot analysis and compared to wildtype cells (wt control) or NTC1 controls. Representative blots of n = 1 biological replicate are shown. Arrow-heads on the left indicate the expected protein

bands. $RIPK3^{-/-}$ and $MLKL^{-/-}$ CRISPR/Cas9 gene knockouts in (C) A549 and (D) H1437 single-cell clones were analyzed by PCR over the sgRNA-targeted exons and compared to wildtype or NTC1 cells. Expected PCR product sizes for the non-mutated exons of wildtype MLKL, or wildtype RIPK3 sequence are indicated in (C, right). Knockout clones selected for subsequent experiments are highlighted in red. M indicates the DNA size marker: 100 bp ladder (NEB); kb = kilobases, bp = base pairs.

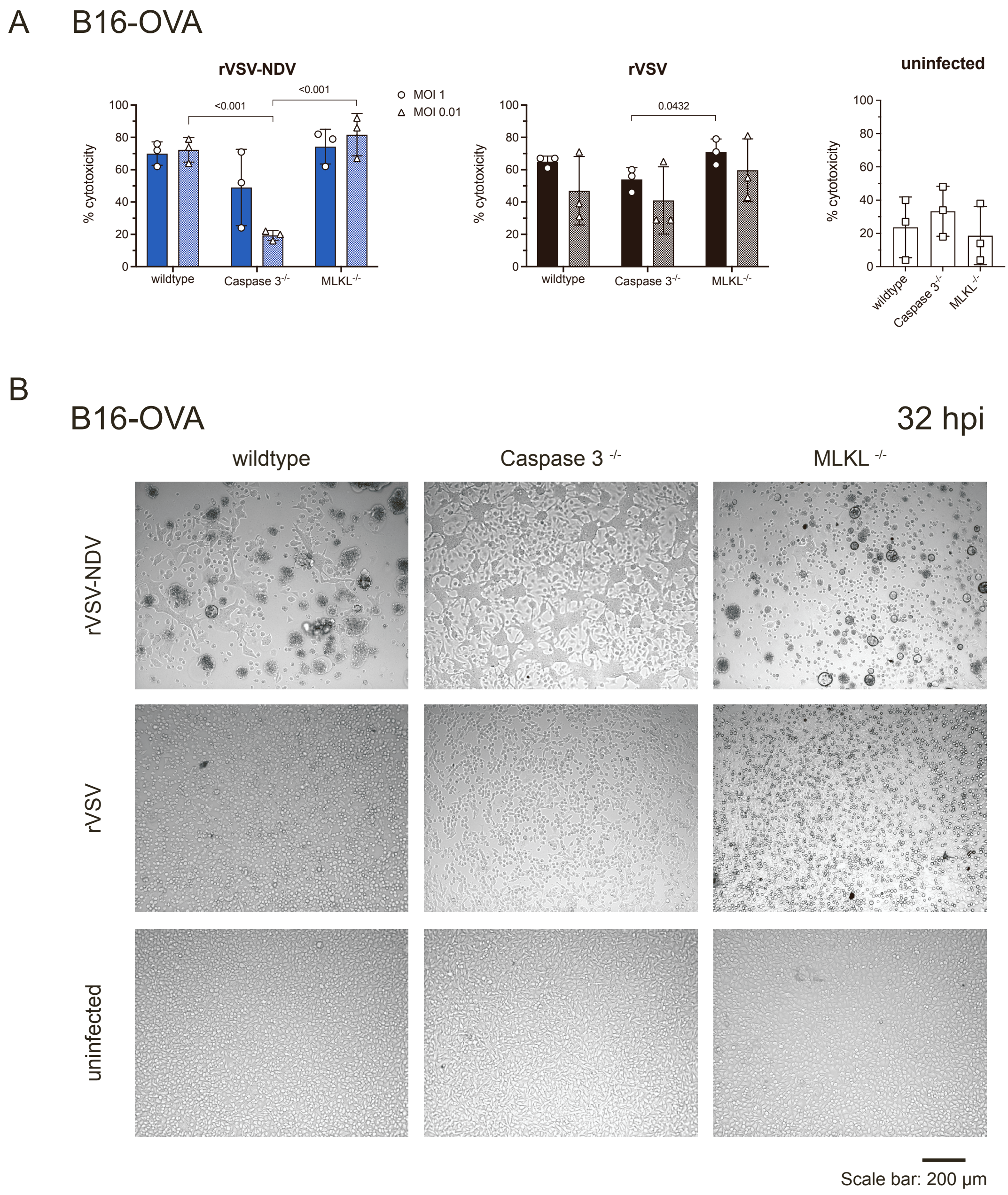

Figure S5: Impact of genetic deletion of downstream apoptosis or necroptosis effectors on rVSV- or rVSV-NDV-mediated oncolysis in B16-OVA cells

B16-OVA wildtype, caspase $3^{-/-}$, or $MLKL^{-/-}$ cells were infected at an MOI of 0.01 with either rVSV-NDV-GFP or rVSV-GFP, or left uninfected. (A) Cell death was measured using the LDH assay at 32 hpi, and data are expressed as the mean % cytotoxicity relative to maximum release control (100 % cytotoxicity) +/- SD values from n = 3 biological replicates. Statistical significance was calculated using one-way ANOVA with Tukey's multiple comparisons test and p values are indicated. (B) Oncolysis progression was recorded at 32 hpi by brightfield

microscopy. Representative images of n = 3 independent experiments and images adjusted to similar contrast and brightness are shown.

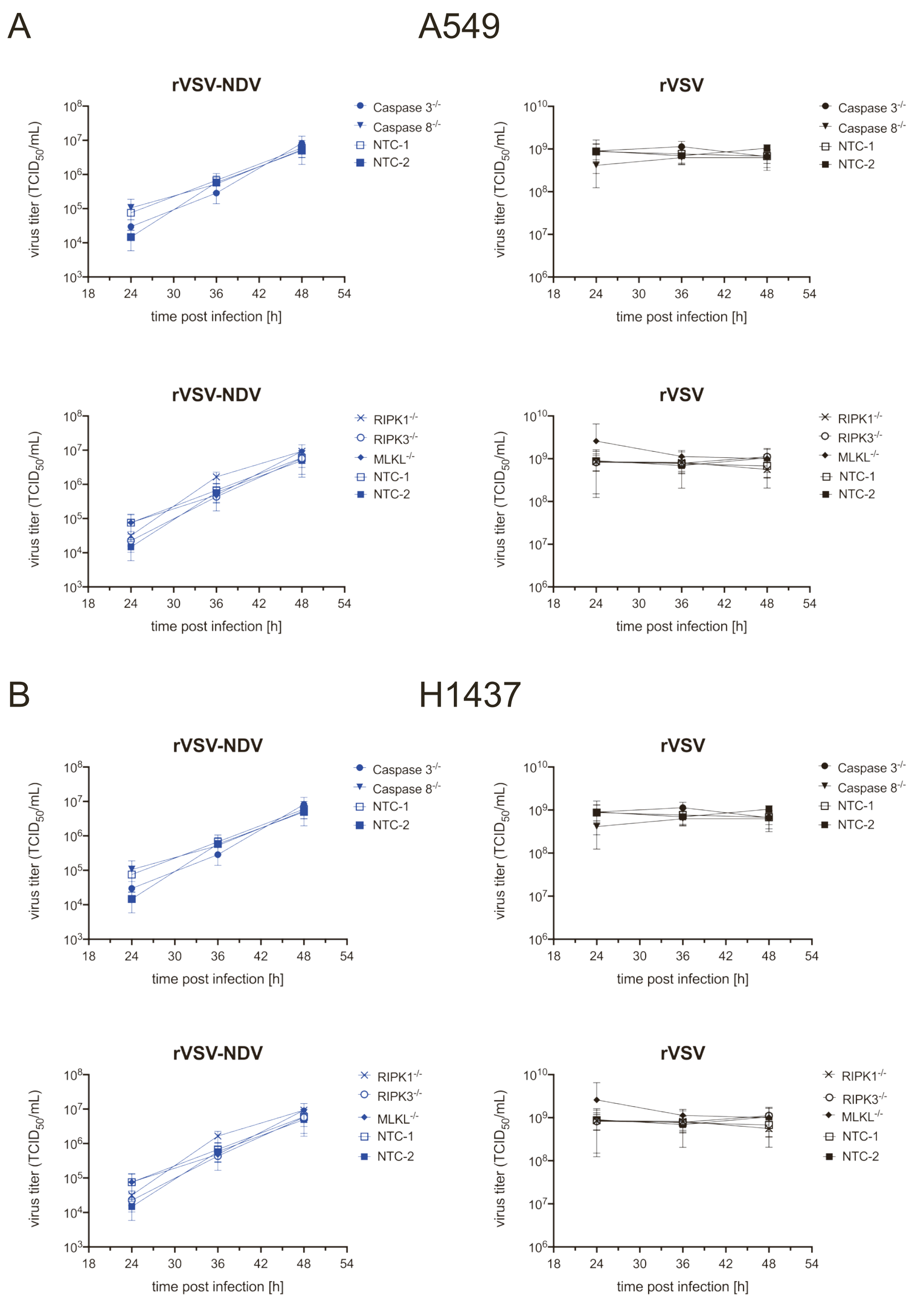

Figure S6: Time course of oncolytic virus replication in A549 and H1437 knockout cell lines

(**A**) A549 and (**B**) H1437 knockout clones were infected at an MOI of 0.01, and changes in viral replication were determined by measuring the extracellular virus concentration at 24, 36, and 48 hpi by $TCID_{50}$ assay. For simplicity, graphs are split into a top panel (caspase $3^{-/-}$ and caspase $8^{-/-}$) and a bottom panel ($RIPK1^{-/-}$, $RIPK^{-/-}$, $MLKL^{-/-}$). Virus concentrations of NTC1 and NTC2 are shown in both panels for better side-by-side comparison. Mean +/- SD values of n = 3 independent biological replicates are shown.

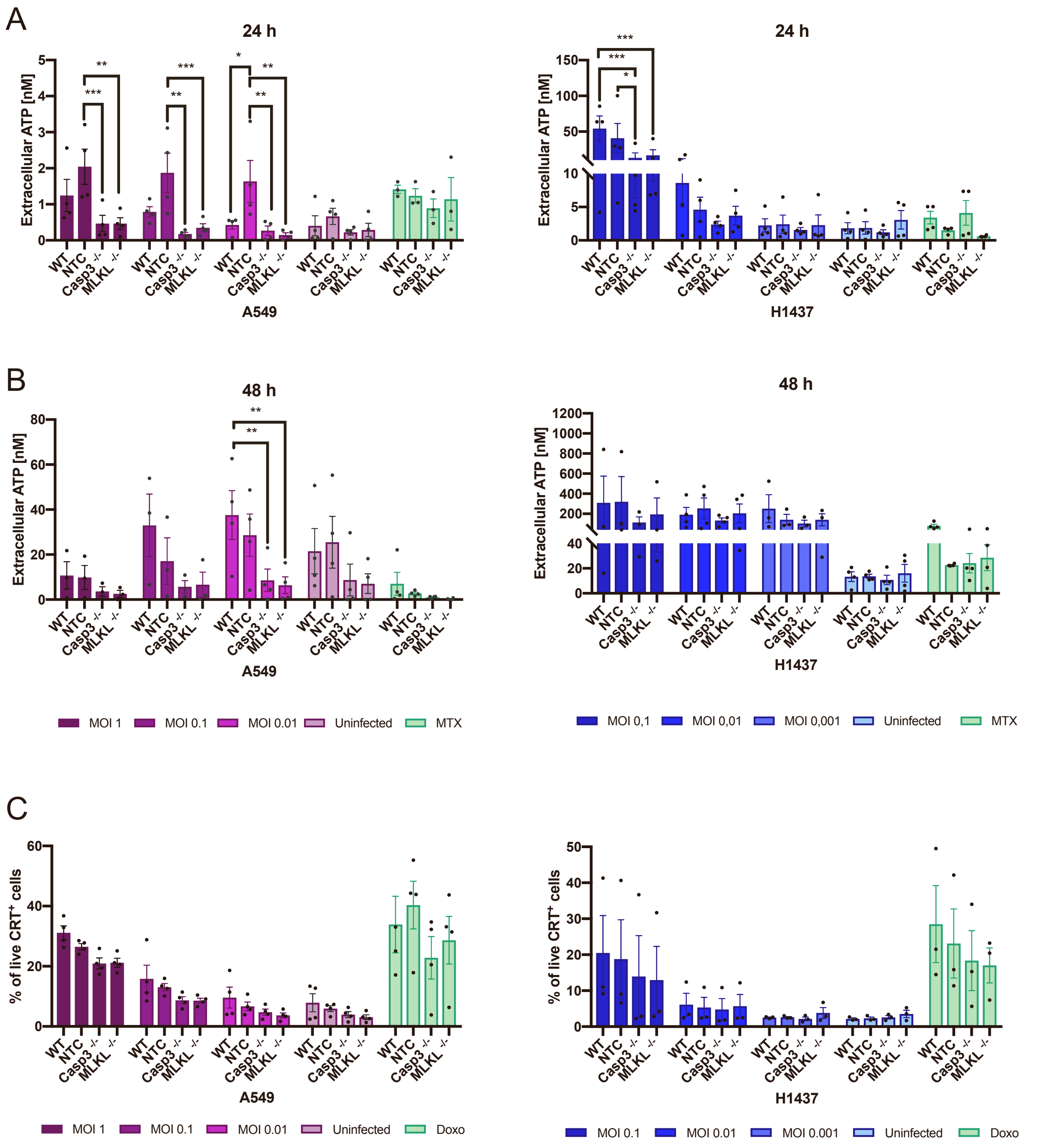

Figure S7. Immunogenic cell death markers in wildtype versus Caspase-3 and MLKL knockout cells. A549 and H1437 cells were infected at a range of MOIs with rVSV-NDV, or treated with MTX or doxorubicin as ICD inducer control. (A) Extracellular ATP quantified from supernatants of OV-infected A549 and H1437 cells at 24 and (B) 48 hpi using a commercial ATP Bioluminescent Assay kit. (C) Flow cytometry-based quantification of calreticulin cell surface exposure in OV-infected A549 (A) and H1437 (D) cells at 24 hpi was calculated as % CRT positive cells among live cells and represented as mean (+/- SD) values of n = 3

independent biological replicates. Statistical significance was tested using one-way ANOVA with Tukey's multiple comparisons test. Plotted are mean (SD) values of n = 4 independent biological replicates.

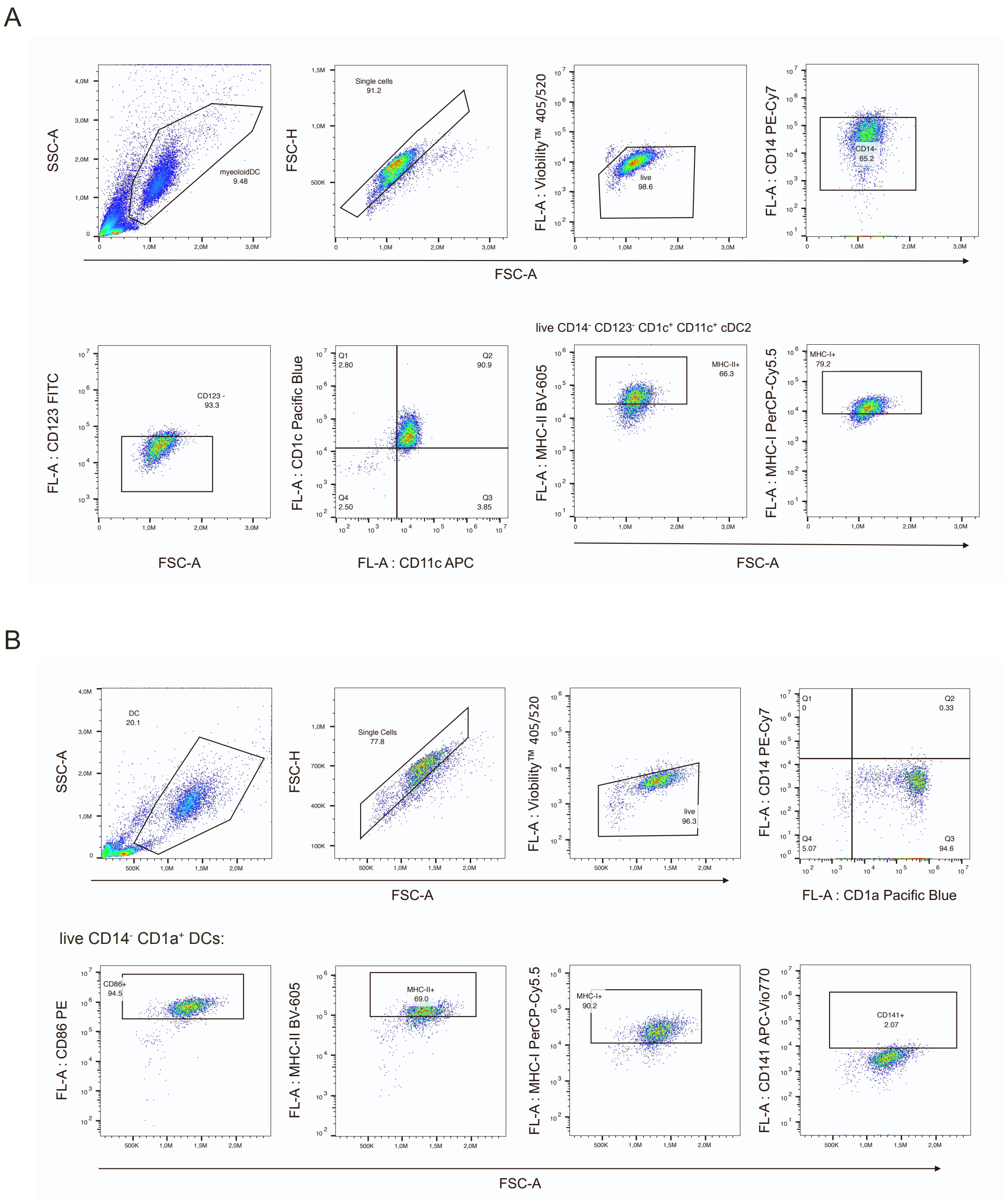

Figure S8: Exemplified gating strategy for *in vitro*-differentiated human cDC2 subtype DCs, and cDC2 activation after cocktail stimulation

(A) Human PBMCs from a healthy donor were analyzed 6-days post GM-CSF + IL-4 stimulation by flow cytometry and characterized as cDC2 subtype cells using antibodies against CD1c, CD11c, CD14, CD123, MHC-I and MHC-II. (B) *In vitro*-differentiated cDC2 cells

(live, CD14$^-$, CD1a$^+$) were cocktail-stimulated, and DC activation (MHC-I, MHC-II, CD86) was measured by flow cytometry. CD141 was used to discriminate cDC2 (CD141$^-$) from cDC1 (CD141$^+$) subtypes. (A and B) Representative scatter plots from FlowJo exemplifying the gating strategy for one human donor each are shown. FSC-A = forward scatter area, SSC-A = sideward scatter area, FSC-H = forward scatter height, FL-A = fluorescence channel area.
